# *Hormophysa cuneiformis* protects against methamphetamine-induced hepatotoxicity via antioxidant, anti-inflammatory, and autophagy-regulatory mechanisms: histological and biochemical studies

**DOI:** 10.3389/fphar.2026.1913493

**Published:** 2026-09-14

**Authors:** Mayada Elhusseiny, Rabab S. El-Kelany, Rania A. El-Shenody, Mohamed S. El-Hashash, Mostafa E. Elshobary, Samar Sami Alkafaas

**Affiliations:** 1 Histology and Cell Biology Department, Faculty of Medicine, Tanta University, Tanta, Egypt; 2 Forensic Medicine and Clinical Toxicology at the Department of Forensic Medicine and Clinical Toxicology, Faculty of Medicine, Tanta University, Tanta, Egypt; 3 Botany and Microbiology Department, Faculty of Science, Tanta University, Tanta, Egypt; 4 Aquaculture Research, AWI-Helmholtz Centre for Polar and Marine Research, Bremerhaven, Germany; 5 Molecular Cell Biology Unit, Division of Biochemistry, Department of Chemistry, Faculty of Science, Tanta University, Tanta, Egypt

**Keywords:** autophagy, brown seaweed, caspase-3, hepatotoxicity, *Hormophysa cuneiformis*, methamphetamine, molecular docking, NF-κB

## Abstract

**Introduction:**

Methamphetamine (METH) abuse is linked to significant hepatic injury, driven by oxidative stress, inflammatory pathway activation, autophagic dysregulation, and apoptosis induction. This study explored the hepatoprotective and anti‐inflammatory potential of the brown seaweed *Hormophysa cuneiformis* extract against METH‐induced liver damage in adult male Wistar rats.

**Methods:**

Extracts prepared using methanol, ethanol, hexane, and water were screened for antioxidant activity. The methanolic extract, selected for further investigation, was characterized by phytochemical analysis and GC–MS profiling. Molecular docking was performed to evaluate the interactions of the identified bioactive compounds with TNF‐α and LC3B‐II. The cytotoxicity and selectivity of the methanolic extract were assessed using HepG‐2 hepatocellular carcinoma and WI‐38 normal cells. Its hepatoprotective effects were further evaluated in vivo in METH‐treated adult male Wistar rats using biochemical, Western blot, immunohistochemical, and histopathological analyses.

**Results:**

The methanolic extract showed the greatest antioxidant activity, achieving 57.16% ± 1.96% DPPH radical scavenging and 54.71% ± 0.84% ferric‐reducing antioxidant power. Although these values were lower than those of ascorbic acid (97.23 ± 0.48%), the methanolic extract exhibited higher antioxidant potential than the other extracts. Secondary metabolites, including alkaloids, saponins, tannins, and total phenolic compounds, were detected in notable amounts. GC‐MS analysis identified oleic acid, palmitic acid, linoleic acid, and arachidonic acid as the major constituents. The extract exhibited dose-dependent cytotoxicity against HepG–2 cells, with an IC50 of 77.14 ± 1.63 µg mL^−1^, while showing lower toxicity toward WI-38 cells (CC50 = 141.99 ± 3.54 µg mL^−1^), indicating selective antiproliferative activity and a favorable safety margin. In vivo, METH administration significantly elevated serum alanine aminotransferase (ALT), aspartate aminotransferase (AST), and alkaline phosphatase (ALP) levels. Oral co-treatment with the *H. cuneiformis* methanolic extract (15 mg kg^−1^) significantly attenuated METH-induced hepatocellular injury, lowering serum ALT from 89.33 to 54.33 U L^−1^, AST from 294.33 to 57.33 U L^−1^, and ALP from 264.67 to 189.00 U L^−1^. Western blot analysis showed that METH markedly increased LC3B-II and Beclin-1 expression, whereas co-administration of the extract significantly reduced both proteins. Immunohistochemical analysis also demonstrated increased caspase-3- and NF-κB-positive cells in the METH-treated group, which were significantly reduced following H. cuneiformis treatment.

**Discussion:**

Collectively, these findings suggest that *H. cuneiformis* exerts hepatoprotective effects against METH-induced liver injury through antioxidant, anti-inflammatory, anti-apoptotic, and autophagy-modulating mechanisms.

## 1 Introduction

Methamphetamine (METH) is a strong central nervous system stimulant widely known for its high abuse potential (Prakash et al., 2017). However, it is approved in limited clinical settings for the treatment of attention deficit hyperactivity disorder (ADHD) and for the short-term management of severe obesity (Åhman, 2024; Akgun et al., 2024). Nonetheless, owing to its strong abuse capacity and systemic toxic effects, methamphetamine misuse has emerged as a serious public health concern. In addition to its well-established neurotoxicity, methamphetamine (METH) has emerged as an important cause of liver injury. Both clinical and experimental studies have demonstrated that METH disrupts bile acid homeostasis and induces hepatotoxicity through multiple interconnected mechanisms, including oxidative stress, excessive reactive oxygen species (ROS) production, lipid peroxidation, activation of inflammatory pathways, cell cycle disruption, and apoptosis (Sovri et al., 2021; Ramli et al., 2025; Omidvari et al., 2023; Rashidi et al., 2024). Further evidence shows that antioxidant compounds, including chlorogenic acid and caftaric acid, can markedly decrease METH-induced oxidative stress and hepatic injury, underscoring the critical involvement of redox-regulated pathways in METH toxicity (Moshiri et al., 2020; Shabani et al., 2023; Wu et al., 2025). Clinical reports further confirm these findings, including cases of acute toxic hepatitis, liver failure, and neonatal cholestasis related to prenatal exposure. The degree of severity ranges from mild, temporary hepatitis to life-threatening liver failure, underscoring the urgent need for effective hepatoprotective approaches (Dahshan, 2009; Maranella et al., 2019; Li et al., 2021; Zhang et al., 2022). On the other hand, seaweeds are widely recognized as an abundant natural source of bioactive compounds that exhibit a broad range of well-established pharmacological properties (Pradhan et al., 2020; Newehy et al., 2025; El Newehy et al., 2026). Traditionally, seaweeds are classified into three main groups, green, red, and brown, each comprising a wide variety of species recognized for their antioxidant, anti-inflammatory, and cytoprotective properties (Ismail et al., 2025; Nguyen and Tran, 2024). Brown seaweeds, in particular, are rich in phenolic compounds, flavonoids, tannins, saponins, alkaloids, and bioactive fatty acids, all of which contribute to their pronounced free-radical-scavenging capacity and hepatoprotective potential (Atya et al., 2021; Remya et al., 2022; El-Sheekh et al., 2025). Previous studies have demonstrated that algal extracts can mitigate liver injury induced by acetaminophen and other hepatotoxic agents by attenuating oxidative stress, modulating inflammatory pathways, and normalizing liver enzyme levels (Sohail et al., 2019; Koshak et al., 2023; Eid et al., 2025). To date, no published studies have examined the capacity of *Hormophysa cuneiformis* to modulate the autophagy-inflammation-apoptosis axis in the context of METH-induced hepatotoxicity. The present study addresses this gap by providing biochemical, molecular, histopathological, and *in silico* evidence for the hepatoprotective potential of this underexplored brown seaweed.

## 2 Materials and methods

### 2.1 Seaweed collection, identification, and extract preparation

Seaweed sample *H. cuneiformis* was collected in January 2024 from Hurghada beach, Egypt. Taxonomic identification was performed according to established morphological keys (Guiry and Nic Dhonncha, 2003; Jha et al., 2009; Belous and Kanaan, 2015; Kanaan et al., 2015), and the species identity was further verified using the Algae Base online database (Mohamed and Saber, 2019; Ahmed and Akl, 2025). Collected samples were carefully rinsed with seawater, followed by tap water, and thereafter delivered to the laboratory under special frozen conditions at 4 °C until analysis. The samples were allowed to air-dry in the dark at ambient temperature, then dried in an oven at 38 °C ± 2 °C. The dried samples were then ground into a fine powder and stored in sealed containers until extraction. The powdered sample was extracted using methanol, ethanol, hexane, and distilled water. Extractions were carried out at a 1:30 (w/v) ratio with continuous agitation at a moderate temperature for 48 h, followed by filtration and solvent evaporation under low heat. The resulting crude extracts were reconstituted to a standardized stock concentration of 5 mg mL^−1^ in the respective extraction solvent and stored at −20 °C until use (Ruzi et al., 2025).

#### 2.1.1 DPPH radical scavenging activity

The DPPH radical scavenging assay was employed to evaluate the electron-donating capacity of the seaweed extracts, based on the method described in Al Zahrani et al. (2020) with minor modifications. Absorbance was measured at 517 nm. The percentage of DPPH radical scavenging activity was calculated using Equation 1:

$$PI\;\left(\%\right)=\left(\;\frac{Ac-AT}{Ac}\;\right)X\;100$$ (1)

Where Ac ​ is the absorbance of the control reaction (2.0 mL of DPPH radical solution without algal extract), and A ​_*T*_ is the absorbance of the test reaction containing the algal extract. The IC_50_ values were calculated using Graphpad Prism.

#### 2.1.2 Ferric-reducing antioxidant power (FRAP)

Ferric-reducing antioxidant power (FRAP) was determined following the procedure described in Marcocci et al. (1994); Ferreira et al. (2007) and Kavak and Basturk (2020). An increase in absorbance reflected a higher reducing capacity of the extract. Ferric-reducing activity was calculated as a percentage relative to the control and expressed as FRAP activity (%).

### 2.2 Antioxidant activity

#### 2.2.1 DPPH radical scavenging activity

The DPPH radical scavenging activity depends on the type of solvent in the antioxidant activity of *H. cuneiformis* extracts (Figure 1). The methanolic extract showed the highest antioxidant activity, with a scavenging percentage of 57.16% ± 1.96%, demonstrating a strong capacity to neutralize DPPH radicals. The ethanolic extract also showed substantial antioxidant activity (52.51% ± 0.702%), while the hexane extract demonstrated moderate activity (43.31% ± 1.65%). On the other hand, the aqueous extract showed relatively low radical scavenging activity (13.64% ± 1.85%). These results confirm that polar solvents, particularly methanol, are more effective at extracting the antioxidant-active constituents of *H. cuneiformis*, consistent with the predominantly phenolic and flavonoid nature of algal antioxidants, and justify the selection of the methanolic extract for subsequent *in vivo* hepatoprotection experiments.

**FIGURE 1:**
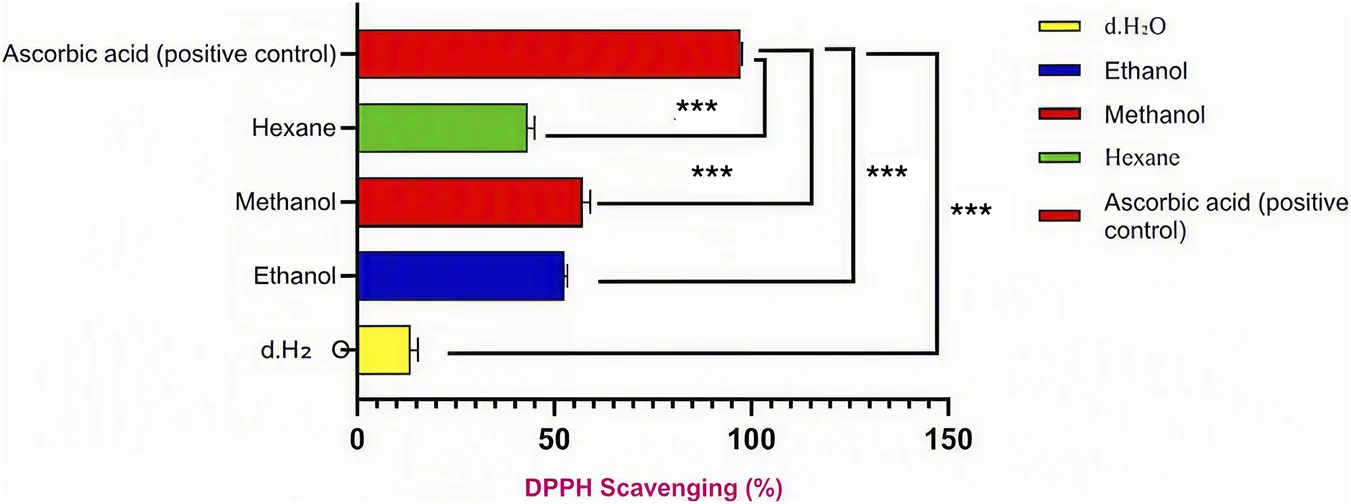
DPPH Radical Scavenging Activity (% Inhibition) of *Hormophysa cuneiformis* Extracts Prepared Using Different Solvents. Values are expressed as mean ± SD (n = 3).

#### 2.2.2 Ferric-*reducing* antioxidant power (FRAP)

The FRAP assay showed a marked variation in the ferric-reducing activity among the different solvent extracts of *H. cuneiformis* (Figure 2). It was observed that the methanolic extract displayed the highest reducing power, demonstrating a FRAP activity of 54.71%, followed by the ethanolic extract (47.77%) and the hexane extract (43.04%). On the other hand, the aqueous extract showed significantly lower activity (8.40%), revealing limited extraction of reductive compounds in H_2_O. Ascorbic acid as expected, was used as a positive control with high activity (96.13%). High FRAP activity was observed for the methanolic and ethanolic extracts of *H. cuneiformis*.

**FIGURE 2:**
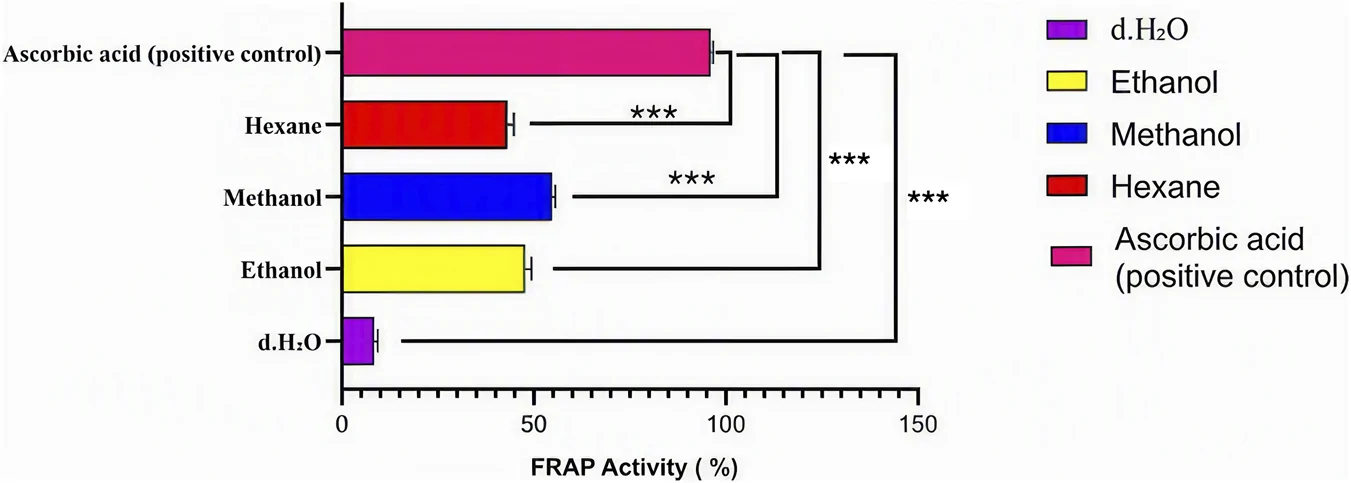
Ferric reducing antioxidant power (FRAP) activity (%) of different solvent extracts (dH_2_O, ethanol, methanol, and hexane) of *Hormophysa cuneiformis*, compared with ascorbic acid as a positive control. Values are expressed as mean ± SD (n = 3).

### 2.3 Phytochemical characterization

#### 2.3.1 Total phenolic, flavonoid, saponin, tannin, and alkaloid content

Total phenolic content was determined according to the method of Taga et al. (1984). Total phenolic content was calculated using a gallic acid standard curve and expressed as milligrams of gallic acid equivalents per gram of crude extract (mg GAE g^−1^ crude extract).

Total flavonoid content was determined using the aluminum chloride colorimetric method as previously described (Chang et al., 2002). Absorbance was measured at 415 nm against a blank in which aluminum chloride was replaced with distilled–deionized water. Total flavonoid content was calculated from a quercetin standard curve and expressed as mg quercetin equivalents per gram of crude extract (mg QE g^−1^ crude extract).

Total saponin content was determined according to Hiai et al. (1975). Total saponin content was calculated using a cholesterol standard curve and expressed as mg cholesterol equivalents per gram of crude extract (mg CE g^−1^ crude extract).

Total tannin content was determined as previously described Shafay et al. (2021). Tannin content was calculated using a tannic acid standard curve and expressed as mg TAE g^-1^ crude extract.

Total alkaloids were quantified following (Harborne, 1998; Fischer et al., 2012). Alkaloids were precipitated by dropwise addition of concentrated ammonia, collected on pre-weighed Whatman No. 1 filter paper, and dried at 70 °C to constant weight. Results were expressed as mg alkaloids per g of crude extract (mg g^−1^ crude extract).

#### 2.3.2 GC–MS profiling of the methanolic extract

Volatile constituents of the algal extract were analyzed using a PerkinElmer Clarus 580/560S GC–MS system equipped with an Elite-5MS capillary column (30 m × 0.25 mm internal diameter, 0.25 μm film thickness), following previously reported procedures (Sparkman et al., 2011; Zheng et al., 2025). Relative quantification was carried out using peak area normalization. The detected compounds were identified based on their retention times and further described according to peak area, peak height, and relative abundance.

### 2.4 Computational analysis

Molecular docking was performed to evaluate binding interactions of GC-MS-identified fatty acid constituents (oleic, palmitic, linoleic, arachidonic, stearic, lauric, myristic acids) and literature-reported phenolic/flavonoid compounds previously documented in *H. cuneiformis* (quercetin, kaempferol, caffeic acid, ferulic acid, gallic acid, ellagic acid, phloroglucinol) against TNF-α (PDB: 2AZ5) and LC3B (PDB: 7ELG). The fatty acid, phenolic, and flavonoid ligands were modeled using software such as AutoDock Vina and Discovery Studio Visualizer, following standard protein preparation (water removal and hydrogen atom addition). Docking was performed using AutoDock Vina and BIOVIA Discovery Studio Visualizer (Alkafaas et al., 2024). All structure minimizations were performed until an RMSD gradient of 0.05 kcal∙mol^−1^Å^−1^ with *MMFF94x* force field, and partial charges were automatically calculated. All intervening water molecules were removed from the structure, and then the target protein was prepared for docking using *Protonate 3D* protocol in AutoDock Vina with default parameters. The co-crystalized ligand was used to define the binding site for docking simulation. The *Triangle Matcher Placement* method and *London dG* scoring function were employed for docking and scoring. The docking protocol was first validated by self-docking the co-crystallized ligand near the protein’s binding site. The ligand-receptor interactions at the protein binding site were studied with the validated docking protocol (RMSD <2) for the reported inhibitors to predict their binding mode and binding affinity.

### 2.5 Cytotoxicity assessment using the MTT assay

The cytotoxic potential of the tested sample was evaluated against HepG-2 (human hepatocellular carcinoma) and WI-38 (normal human lung fibroblast) cell lines. Both cell lines were obtained from the American Type Culture Collection (ATCC, United States of America). HepG-2 cells were maintained in RPMI-1640 medium supplemented with 10% heat-inactivated fetal bovine serum (FBS) and 50 µg mL^−1^ gentamycin, whereas WI-38 cells were cultured in DMEM containing 10% heat-inactivated FBS, 1% L-glutamine, HEPES buffer, and 50 µg mL^−1^ gentamycin. All cells were incubated at 37 °C in a humidified atmosphere of 5% CO_2_. For the cytotoxicity assay, HepG-2 cells were seeded at 5 × 10^4^ cells/well and WI-38 cells at 1 × 10^4^ cells/well in 96-well plates and allowed to adhere for 24 h. Serial dilutions of the tested sample (ranging from 1000 to 2 µg mL^−1^) were added in triplicate wells, while untreated cells and 0.5% DMSO were used as controls. After 48 h of incubation with the sample, cell viability was determined using the MTT colorimetric assay as described by Mosmann (1983). Briefly, wells were supplied with 10 µL MTT solution (12 mM) and incubated for 4 h at 37 °C. The resulting formazan crystals were dissolved by adding 50 µL DMSO, and absorbance was measured at 590 nm using a TECAN microplate reader. Cell viability was calculated using Equation 2:

$$\text{Viability }\%=\left(\frac{{\text{OD}}_{t}}{{\text{OD}}_{c}}\right)\times 100$$ (2)

Where *ODt* represents the absorbance of treated wells and *ODc* the absorbance of control wells. The IC_50_ for HepG-2 and CC_50_ for WI-38 were determined from the dose–response curves using GraphPad Prism software.

### 2.6 *In Vivo* study design and experimental protocol

Forty adult male Wistar albino rats (150–200 gm) were housed in stainless steel cages under controlled conditions (23 °C ± 2 °C, 55%–60% humidity, 12 h light/dark cycle) with free access to standard pellet diet and water. Baseline blood glucose levels were recorded before the experiment. All procedures were conducted in accordance with the Institutional Animal Care and Use Committee guidelines, Faculty of Science, Tanta University (IACUC-SCI-TU-0437).

This study was carried out on 40 adult male albino rats. They were divided into 4 main groups as follows:

Group I (control group): included 10 rats that were subdivided equally into 2 subgroups:

Subgroup Ia: included 5 rats. Each rat received 1 mL of saline as a vehicle (once daily) orally for 10 days.

Subgroup Ib: included 5 rats. Each rat received 1 mL of saline by intraperitoneal injection (once daily) for 7 days. Both subgroups showed comparable results and were therefore treated as a single control group of ten rats.

Group II: included 10 rats. Each rat received 1 mL saline containing 5 mg kg^−1^ of Methamphetamine (METH) daily intraperitoneally for 7 days. Methamphetamine was obtained from Desoxyn® tablets (methamphetamine hydrochloride; [Keyrx, United States of America]) and freshly dissolved in sterile saline prior to administration (Jing et al., 2014; Zhang et al., 2022).

Group III: included 10 rats which received 1 mL of saline containing 15 mg kg^−1^ methanolic extract of *H. cuneiformis* daily orally for 10 consecutive days (Jatav et al., 2014).

Group IV: included 10 rats which received *H. cuneiformis* methanolic extract for 3 consecutive days before injection of METH and concomitantly with METH for another 7 days. Both *H. cuneiformis* methanolic extract and METH were administered at the same previous dose and route.

At the end of the experimental period, animals were deeply anesthetized using isoflurane inhalation (5% isoflurane in oxygen delivered via an induction chamber) until loss of pedal withdrawal and corneal reflexes was confirmed. Animals were then euthanized under deep anesthesia, and blood samples were collected by cardiac puncture followed by tissue harvesting. Rats in subgroup Ia as well as groups III and IV were sacrificed on day 10, whereas those in subgroup Ib and group II were sacrificed on day 7. Blood samples were obtained via cardiac puncture and then centrifuged at 3000 rpm for 20 min at 4 °C to separate the serum.

The livers were carefully removed, rinsed with saline, and divided into two parts. One portion was stored at −80 °C for subsequent protein extraction and Western blot analysis. The other portion was fixed in 10% formalin for histopathological and immunohistochemical examinations.

#### 2.6.1 Liver function enzymes

Overall, enzymatic and oxidative stress assays were accomplished according to kit instructions and spectrophotometric methods:

For serum alanine aminotransferase (ALT) determination, four parts of R1 (Good’s buffer, L-alanine, LDH) were mixed with one part of R2 (NADH, oxoglutarate), while for aspartate aminotransferase (AST), four parts of R1 (phosphate buffer, L-aspartate) were mixed with one part of R2 (LDH, MDH, NADH, oxoglutarate). Serum (200 µL) was added to 2 mL of the working reagent, and absorbance at 340 nm was recorded every minute for 3 min at 37 °C. Enzyme activity was calculated as Equation 3 ALT or AST (U L^−1^) = ΔA/min × 1746 (Knight, 2005).

For alkaline phosphatase (ALP) determination, the reaction was carried out at 37 °C, and the change in absorbance per minute (ΔA/min) was measured at 405 nm, following the method described by (Tang et al., 2019). Alkaline phosphatase (ALP) activity was calculated using the Equation 4 ALP (U L^−1^) = ΔA/min × 545(4).

#### 2.6.2 Molecular analysis (Western blot)

For Western blot analysis, liver tissues were homogenized in ice-cold RIPA buffer supplemented with 1% protease inhibitor cocktail (Santa Cruz Biotechnology; Cat. no. sc-29130) and phosphatase inhibitor cocktail for phosphorylated targets (Santa Cruz Biotechnology; Cat. no. sc-45044). Total protein concentration was determined using the bicinchoninic acid (BCA) assay (Santa Cruz Biotechnology; Cat. no. sc-278767A), followed by denaturation in 4× Laemmli buffer. Equal amounts of protein were separated on 10% SDS-PAGE and wet-transferred onto PVDF membranes using a Mini Trans-Blot® Cell (Bio-Rad, Hercules, CA, United States of America). Membranes were blocked for 1 h at room temperature in 5% BSA/TBST and incubated overnight at 4 °C with primary antibodies diluted in 3% BSA/TBST. Primary antibodies included mouse monoclonal anti-β-actin (C4; 1:1000; Santa Cruz Biotechnology; Cat. No. sc-47778) as a loading control, mouse monoclonal anti-LC3B-II (G-2; 1:1000; Santa Cruz Biotechnology; Cat. No. sc-271625), and mouse monoclonal anti-Beclin-1 (E-8; 1:1000; Santa Cruz Biotechnology; Cat. No. sc-48341). After washing, membranes were incubated for 1 h at room temperature with alkaline phosphatase-conjugated anti-mouse secondary antibody (1:10,000; SeraCare, Milford, MA, United States of America). Protein bands were visualized using NBT/BCIP substrate (Thermo Fisher Scientific, Waltham, MA, United States of America), quantified using Image Studio Lite software (LI-COR Biotechnology, Lincoln, NE, United States of America), and normalized to β-actin (Jiang et al., 2012).

#### 2.6.3 Histopathological examination

Specimens from livers were taken and immediately fixed in 10% formal saline solution for 24 h, dehydrated in ascending grades of alcohol, and then cleared in two changes of xylol. Impregnation was performed in pure soft paraffin for 2 h at 55 °C, followed by embedding in hard paraffin. Finally, paraffin sections (5 µm thick) were cut using a microtome, mounted on glass slides, and stained with hematoxylin and eosin (H&E) according to the standard protocol described by Bancroft and Gamble (2008). The stained sections were examined and photographed using a light microscope.

#### 2.6.4 Immunohistochemical staining

Immunohistochemical staining for caspase-3 and nuclear factor kappa B was carried out following the procedures described by Bancroft and Gamble (2008) and Sharma and Gandhi (2012), using the avidin–biotin–peroxidase method. Sections were deparaffinized by putting slides in xylol for 30 min. Sections were then rehydrated through descending grades of alcohol (5 min each), followed by distilled water for 5 min. Sections were placed in 0.3% hydrogen peroxide/methanol for 30 min to reduce nonspecific background staining due to endogenous peroxidase. After that, sections were washed in phosphate buffer saline for 5 min. Antigen retrieval was done by boiling the section in 10 mM sodium citrate (PH 6.0) for 10–20 min followed by cooling at room temperature for 20 min. To prevent nonspecific binding, the sections were incubated in 5% human serum albumin (blocking protein) for 30min followed by washing three times in phosphate-buffered saline. The sections were then incubated overnight in a humid chamber at 4 °C with primary antibodies (anti-caspase monoclonal antibody or anti-NF-κB, dilution 1: 400–800, obtained from Lab Vision Company). Slides were then washed three times in PBS, 10 min each. Biotinylated Goat Anti-polyvalent was applied for 10 min at room temperature, and slides were then washed in phosphate-buffered saline. Slides were incubated for one hour with peroxidase-conjugated secondary antibodies (peroxidase-labelled streptavidin, dilution 1:200) obtained from Sigma Chemical Company. The reaction was visualized with 0.05% diaminobenzidine (DAB) as a chromogen and 0.005% H_2_O_2_ as substrate. Finally, slides were counterstained with hematoxylin, then dehydrated, cleared, and mounted for microscopic evaluation.

### 2.7 Statistical analysis

Data are expressed as mean ± standard deviation (SD). Group differences were analyzed using one-way ANOVA followed by Tukey’s *post hoc* test for multiple comparisons, conducted with GraphPad Prism (version 10.0). The number of biological replicates (n) for each experiment is indicated in the corresponding figure legends. A p-value <0.05 was considered statistically significant.

## 3 Results

### 3.1 Phytochemical characterization

The phytochemical constituents of *H. cuneiformis* were investigated using several solvents, including methanol, ethanol, hexane, and water (Table 1).

**TABLE 1:** Phytochemical constituents of *H. cuneiformis* extracts.

Solvent	Extraction yield (mg g^−1^ DW)	Total phenols (mg GAE g^−1^ crude extract)	Flavonoids (mg QE g^−1^ crude extract)	Saponins (mg CE g^−1^ crude extract	Tannin (mg TAE g^−1^ crude extract)	Alkaloids (mg g^−1^ crude extract)
Methanol	138	2.02 ± 0.61^b^	1.01 ± 0.06^a^	15.19 ± 0.33^a^	3.42 ± 0.82^b^	19.83 ± 2.25 ^ab^
Ethanol	51	3.01 ± 1.11^b^	1.04 ± 0.04^a^	13.41 ± 2.99^ab^	1.35 ± 0.78^d^	21.10 ± 0.92^a^
Hexane	15	3.59 ± 1.75^b^	1.13 ± 0.09^a^	12.97 ± 1.31^b^	2.40 ± 0.42^c^	16.13 ± 2.36^b^
H2O	255	13.37 ± 1.49^a^	0.11 ± 0.01^b^	6.04 ± 0.44^c^	8.42 ± 0.98^a^	0.55 ± 0.14^c^

Different superscript letters in each column indicate significant differences at p < 0.05.

#### 3.1.1 Secondary metabolite quantification

Aqueous extract of *H. cuneiformis* showed a high total phenolic content with 13.37 ± 1.49 mg GAE g^−1^ crude extract, indicating phenolic compounds are more efficiently extracted in water. The hexane extract contained 3.59 ± 1.75 mg GAE g^-1^ crude extract, followed by the ethanolic extract at 3.01 ± 1.11 mg GAE g^-1^ crude extract, while the methanolic extract showed the lowest total phenolic content (2.02 ± 0.61 mg GAE g^-1^ crude extract) (Table 1). It was observed that the flavonoid content of *H. cuneiformis* remained stable among the organic solvent extracts. Methanolic extract (1.01 ± 0.06 mg QE g^-1^ crude extract) and ethanolic extract (1.04 ± 0.04 mg QE g^-1^ crude extract) showed similar values of flavonoid content. Hexane extract was observed to have slightly higher flavonoid concentration (1.13 ± 0.09 mg QE g^-1^ crude extract). On the other hand, the aqueous extract displayed a significantly lower level, with only 0.11 ± 0.01 mg QE g^-1^ crude extract detected (Table 1).

Table 1 shows the total saponins content of *H. cuneiformis*. The variable total saponins content depends on the extraction solvent. It was observed that the methanolic extract exhibited the highest saponins concentration (15.19 ± 0.33 mg CE g^−1^ crude extract), followed by the ethanolic extract (13.41 ± 2.99 mg CE g^−1^ crude extract). On the other hand, the hexane extract yielded a moderately high saponins level (12.97 ± 1.31 mg CE g^−1^ crude extract), and the aqueous extract had the lowest content (6.04 ± 0.44 mg CE g^−1^ crude extract). Collectively, organic solvents, especially methanol and hexane, were more effective at extracting saponins from *H. cuneiformis*.

Table 1 shows the total tannin content of *H. cuneiformis*. The total tannin content varies depending on the different extraction solvents. It was observed that the aqueous extract displayed the highest tannin concentration (8.42 ± 0.98 mg TAE g^−1^ crude extract), indicating that tannins in *H. cuneiformis* are largely water-soluble. On the other hand, methanol extraction yielded a moderate tannin level (3.42 ± 0.82 mg TAE g^−1^ crude extract), and the hexane extract contained a lower amount (2.40 ± 0.42 mg TAE g^−1^ crude extract). Also, it was observed that the ethanolic extract showed the lowest tannin content (1.35 ± 0.78 mg TAE g^−1^ crude extract).

The alkaloids content of *H. cuneiformis* varied significantly according to the extraction solvent. The methanolic extract had 19.83 ± 2.25 mg g^−1^ crude extract, while the ethanolic extract showed a slightly higher level (21.10 ± 0.92 mg g^−1^ crude extract). The hexane extract had a lower alkaloids concentration (16.13 ± 2.36 mg g^−1^ crude extract). On the other hand, the aqueous extract showed a significantly lower alkaloids content (0.55 ± 0.14 mg g^−1^ crude extract). Taken together, these data indicate that organic solvents were more effective for extracting alkaloids and flavonoids from *H. cuneiformis*, whereas water was found to be the most efficient solvent for extracting phenolic compounds (Table 1).

#### 3.1.2 GC–MS analysis

Figure 3 and Table 2 show the GC–MS results of the methanolic extract of *H. cuneiformis*. The total ion chromatogram showed that lipids are predominant in nature, with a mixture of fatty acids, terpenoid compounds, and sterol-related structures. The most intense peak appeared at a retention time (RT) of 28.904 min and was assigned to cis-vaccenic/oleic acid, which demonstrated the highest relative abundance (Area% = 16.877; Norm% = 100%). Palmitic acid was the next major constituent, identified at RT 25.477 min with an Area% of 12.653. An obvious peak in the early retention-time region (RT 9.031 min) corresponded primarily to squalene and related isoprenoid compounds, indicating a significant terpenoid in the crude extracts. Dodecanoic (lauric) acid was also present at RT 17.439 min, while in a lower proportion (Area% = 1.398%).

**FIGURE 3:**
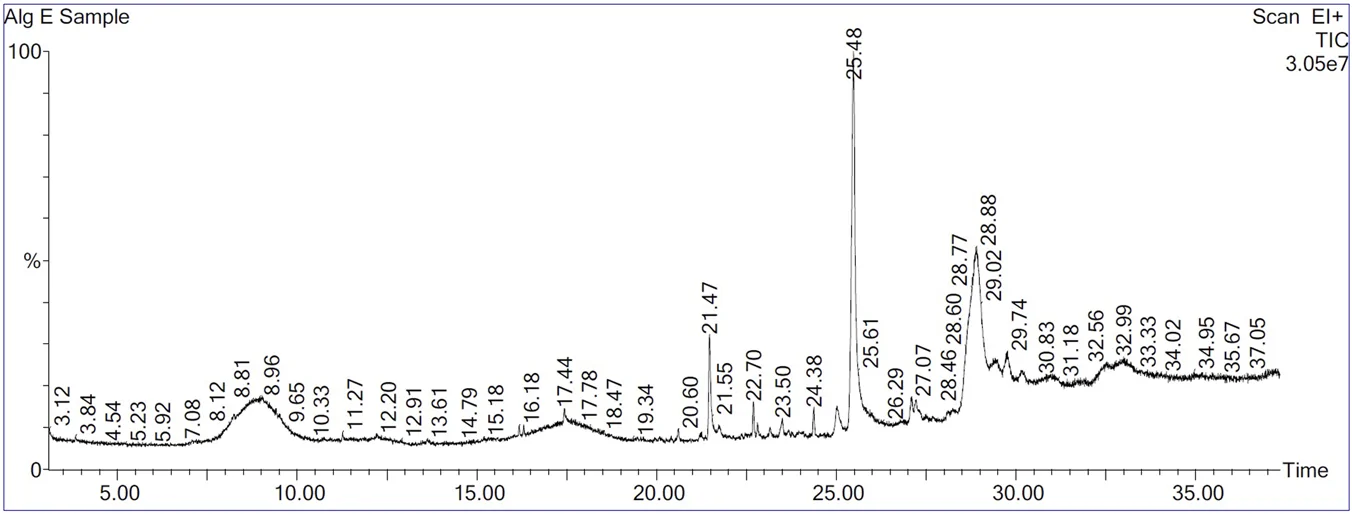
Total ion chromatogram (TIC) of the algal extract obtained by GC–MS analysis.

**TABLE 2:** Gas chromatography-mass spectrometry (GC–MS) peak profile of the analyzed sample, showing retention time (RT), peak area, and molecular formula.

RT (min)	Compound name	PA%	MF
8.245	Squalene	1.831	C_30_H_50_
9.031	Squalene	10.573	C_30_H_50_
9.421	10-Methyl-E-11-tridecen-1-ol propionate	2.805	C_17_H_30_O_2_
17.254	Stigmastan-3,5-diene	1.066	C_29_H_48_
17.439	Dodecanoic acid	1.398	C_12_H_24_O_2_
21.471	Myristic acid	2.055	C_14_H_28_O_2_
24.377	Hexadecanoic acid, methyl ester	0.365	C_17_H_34_O_2_
25.477	Palmitic acid	12.653	C_16_H_32_O_2_
28.904	cis-Vaccenic acid	16.877	C_18_H_34_O_2_
29.414	Octadecanoic acid	3.699	C_18_H_36_O_2_
29.764	Arachidonic acid	2.899	C_20_H_32_O_2_
30.159	5,8,11,14-Eicosatetraenoic acid, methyl ester	2.125	C_21_H_34_O_2_

The fatty acid composition involved both saturated and unsaturated fatty acids. Saturated fatty acids, such as lauric, myristic, palmitic, and stearic acids, were identified alongside unsaturated fatty acids including oleic acid, linoleic acid (identified as its methyl ester), and arachidonic acid. These findings were confirmed by strong spectral matching. In the mid-retention time region, various sesquiterpenes, diterpenoid alcohols, and sterol derivatives were observed, including remarkably stigmastan-3,5-diene and sitosterol acetate. Their existence is consistent with the occurrence of bioactive secondary metabolites commonly reported in lipid-rich algal extracts. Furthermore, various minor constituents, such as long-chain hydrocarbons, methyl esters, and oxygenated terpenoids, were detected in trace amounts, contributing to the overall chemical diversity of the extract.

### 3.2 Computational analysis

Molecular docking was performed for the fatty acids as ligands, phenolic and flavonoid as ligands involved in *H. cuneiformis* against several proteins, including the crystal structure of TNF-α with a small molecule inhibitor (PDB: 2AZ5), and LC3B-II modified with a covalent probe (PDB:7ELG), as shown in Table 3 and Figures 4–7. Figures 4, 5 illustrate the 3D binding poses and 2D interaction profiles of the tested compounds with TNF-α. Fatty acids were stabilized mainly through hydrophobic contacts, whereas phenolic and flavonoid compounds formed hydrogen bonds and π-interactions with key binding-site residues. Similarly, Figures 6, 7 demonstrate that the compounds occupied the LC3B-II binding pocket through combinations of hydrophobic, hydrogen-bonding, electrostatic, and π-mediated interactions. These interaction patterns support the predicted docking scores and binding affinities. Moreover, the docked natural ligands were compared with the reference inhibitor (pomalidomide) for TNF-α and hydroxychloroquine for LC3B-II to provide a relative assessment of the predicted binding affinities of the tested compounds. For TNF-α, the control compound pomalidomide demonstrated a docking score of −5.21 kcal mol^−1^, while the co-crystallized ligand displayed a significantly stronger interaction at −7.11 kcal mol^−1^, indicating that the docking procedure successfully reproduced a favorable binding pose. All major fatty acids exhibited better binding energies compared to the reference drug. Palmitic acid scored −5.82 kcal mol^−1^, stearic acid −6.14 kcal mol−1, oleic acid −6.31 kcal mol^−1^, linoleic acid −6.08 kcal mol^−1^, lauric acid −5.30 kcal mol^−1^, myristic acid −5.67 kcal mol^−1^, and arachidonic acid −6.25 kcal mol^−1^. These docking scores suggest more favorable predicted binding energies than pomalidomide. Among the phenolic compounds, caffeic acid (−4.81 kcal mol^−1^), ferulic acid (−4.93 kcal mol^−1^), phloroglucinol (−3.03 kcal mol^−1^), gallic acid (−4.13 kcal mol^−1^), and ellagic acid (−4.85 kcal mol^−1^) bound less effectively than the reference drug. On the other hand, quercetin (−5.38 kcal mol^−1^) displayed a slightly more favorable predicted docking score compared with pomalidomide, while kaempferol (−4.96 kcal mol^−1^) remained slightly weaker. In summary, only quercetin produced a more favorable docking score than the reference compound among phenolics, whereas several fatty acids showed consistently favorable predicted binding to TNF-α.

**TABLE 3:** Docking scores and binding interactions of fatty acids and polyphenols with TNF-α and LC3B relative to standard drug controls.

Crystal structure of TNF-α with a small molecule inhibitor (PDB:2AZ5)		
Major fatty acids		
Compound/drug	Docking score (kcal mol−1)	Amino acids involved in binding
Co-crystalized ligand	−7.1127 kcal mol^−1^	6-ring TYR^59^ (B) H-pi CB TYR^59^ (A) pi-H
Pomalidomide (control)	−5.2172 kcal mol^−1^	CD2 TYR^119^ (B) pi-H
Palmitic acid (C16:0)	−5.8207 kcal mol^−1^	OH TYR^151^ (A) H-acceptor
Stearic acid (C18:0)	−6.1447 kcal mol^−1^	OH TYR^151^ (A) H-acceptor
Oleic acid (C181, cis-9-octadecenoic acid)	−6.3185 kcal mol^−1^	N SER^95^ (B) H-acceptor
Linoleic acid (C18:2, cis-9,12-octadecadienoic acid)	−6.0844 kcal mol^−1^	N SER^95^ (B) H-acceptor
Lauric acid	−5.3058 kcal mol^−1^	OH TYR^151^ (A) H-acceptor
Myristic acid	−5.6762 kcal mol^−1^	N GLY 121 (B) H-acceptor
Arachidonic acid	−6.2518 kcal mol^−1^	OH TYR 151 (A) H-acceptor
Phenolic compounds		
Caffeic acid	−4.8107 kcal mol^−1^	OH TYR^151^ (A) H-acceptor
Ferulic acid	−4.9265 kcal mol^−1^	OH TYR^151^ (A) H-acceptor
Phloroglucinol (base unit of phloroTannin)	−3.0321 kcal mol^−1^	OH TYR^151^ (A) H-acceptor OH TYR^151^ (A) H-acceptor
Flavonoids		
Kaempferol	−4.9631 kcal mol^−1^	O LEU^120^ (B) H-donor
Quercetin	−5.3891 kcal mol^−1^	CB TYR^59^ (B) pi-H
Gallic acid (3,4,5-trihydroxybenzoic acid)	−4.1378 kcal mol^−1^	O GLY^121^ (A) H-donor
Ellagic acid	−4.8563 kcal mol^−1^	OH TYR^151^ (A) H-acceptor
LC3B modificated with a covalent probe (pdb: 7ELG)		
Major fatty acids		
Co-crystalized ligand	−8.8135 kcal mol−1	N LEU 57 (A) H-acceptor
Hydroxychloroquine (control)	−4.5699 kcal mol−1	N LEU 57 (A) H-acceptor CA VAL 58 (A) pi-H
Palmitic acid (C16:0)	−5.5860 kcal mol^−1^	NZ LYS^53^ (A) H-acceptor NZ LYS^53^ (A) ionic NZ LYS^55^ (A) ionic
Oleic acid (C18:1, cis-9-octadecenoic acid)	−5.3936 kcal mol^−1^	NZ LYS^55^ (A) H-acceptor NZ LYS^55^ (A) ionic NZ LYS^53^ (A) ionic
Linoleic acid (C18:2, cis-9,12-octadecadienoic acid)	−5.4985 kcal mol^−1^	NZ LYS^55^ (A) H-acceptor NZ LYS^55^ (A) ionic NZ LYS^53^ (A) ionic
Stearic acid (C18:0)	−5.4298 kcal mol^−1^	NZ LYS^55^ (A) H-acceptor NZ LYS^53^ (A) H-acceptor NZ LYS^53^ (A) ionic NZ LYS^55^ (A) ionic
Lauric acid	−5.2053 kcal mol^−1^	N LEU^57^ (A) H-acceptor NZ LYS^53^ (A) ionic
Myristic acid	−5.4349 kcal mol^−1^	NZ LYS^53^ (A) H-acceptor NZ LYS^55^ (A) H-acceptor NZ LYS^53^ (A) ionic NZ LYS^55^ (A) ionic
Arachidonic acid	−5.8656 kcal mol^−1^	NZ LYS^53^ (A) H-acceptor N LEU^57^ (A) H-acceptor NZ LYS^53^ (A) ionic
Phenolic compounds		
Caffeic acid	−4.2489 kcal mol^−1^	NZ LYS^55^ (A) H-acceptor NZ LYS^55^ (A) ionic NZ LYS^53^ (A) ionic NZ LYS^55^ (A) ionic
Ferulic acid	−4.4064 kcal mol^−1^	NZ LYS^55^ (A) H-acceptor NZ LYS^55^ (A) ionic NZ LYS^53^ (A) ionic NZ LYS^55^ (A) ionic
Phloroglucinol	−4.2852 kcal mol^−1^	NH1 ARG^74^ (A) H-acceptor N LEU^57^ (A) H-acceptor NE ARG^74^ (A) ionic NH1 ARG^74^ (A) ionic NZ LYS^53^ (A) ionic NH1 ARG^74^ (A) ionic NZ LYS^53^ (A) ionic
Flavonoids		
Kaempferol	−5.0513 kcal mol^−1^	N LEU^57^ (A) H-acceptor NZ LYS^53^ (A) ionic
Quercetin	−4.9706 kcal mol^−1^	N LEU^57^ (A) H-acceptor NZ LYS^53^ (A) ionic
Ellagic acid	−5.1673 kcal mol^−1^	NZ LYS^34^ (A) H-acceptor NZ LYS^55^ (A) H-acceptor NZ LYS^34^ (A) ionic NZ LYS^53^ (A) ionic NZ LYS^55^ (A) ionic
Gallic acid (3,4,5-trihydroxybenzoic acid)	−4.1777 kcal mol^−1^	NZ LYS^53^ (A) H-acceptor N LEU^57^ (A) H-acceptor NZ LYS^53^ (A) ionic

**FIGURE 4:**
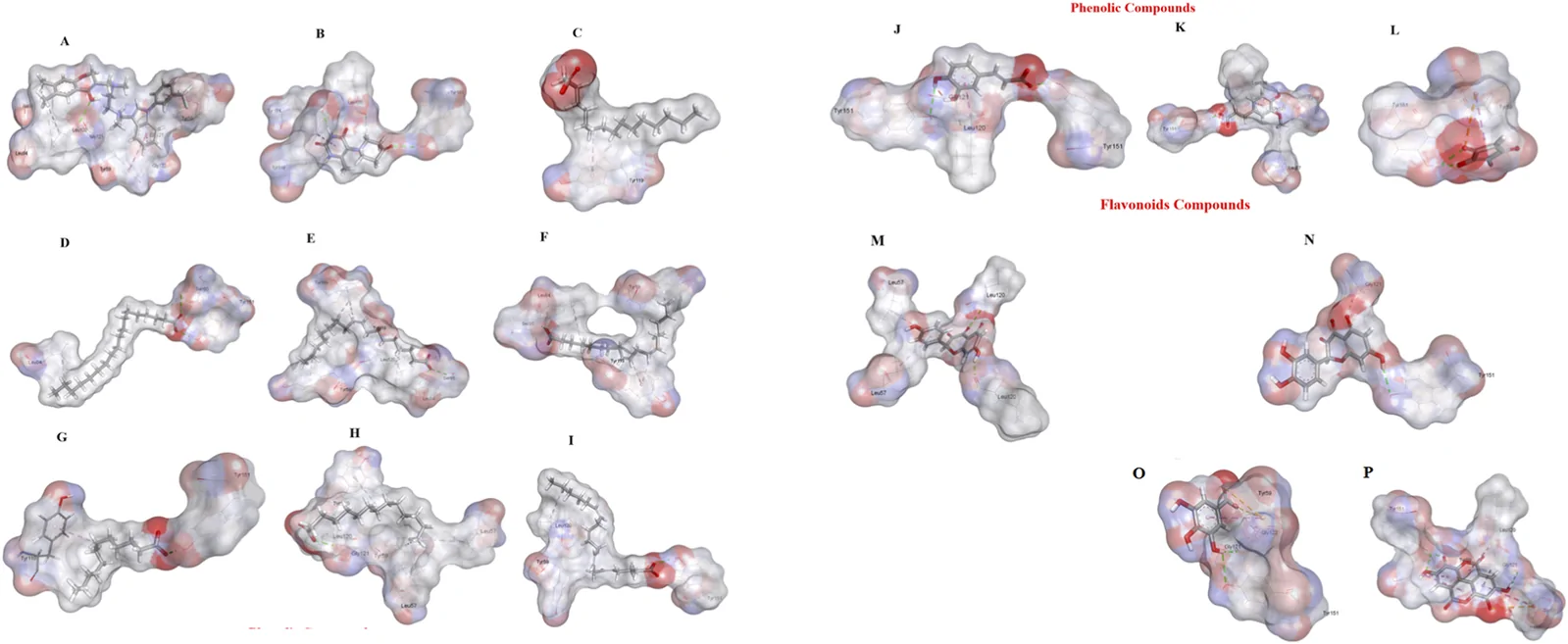
Three-dimensional molecular docking visualization of the binding modes of the co-crystallized ligand and the major phytochemical constituents identified in the extract within the active site of the target protein. The protein is displayed as a semi-transparent molecular surface colored according to electrostatic potential, whereas the docked ligands are shown in stick representation. The colored dashed lines indicate the different protein–ligand interactions: green, conventional hydrogen bonds; cyan, carbon–hydrogen bonds; pink, alkyl and π–alkyl interactions; purple, π–π and π–σ interactions; and orange, electrostatic interactions. Panels represent: **(A)** co-crystallized ligand, **(B)** pomalidomide, **(C)** palmitic acid, **(D)** stearic acid, **(E)** oleic acid, **(F)** linoleic acid, **(G)** lauric acid, **(H)** myristic acid, **(I)** arachidonic acid, **(J)** caffeic acid, **(K)** ferulic acid, **(L)** phloroglucinol, **(M)** kaempferol, **(N)** quercetin, **(O)** gallic acid, and **(P)** ellagic acid.

**FIGURE 5:**
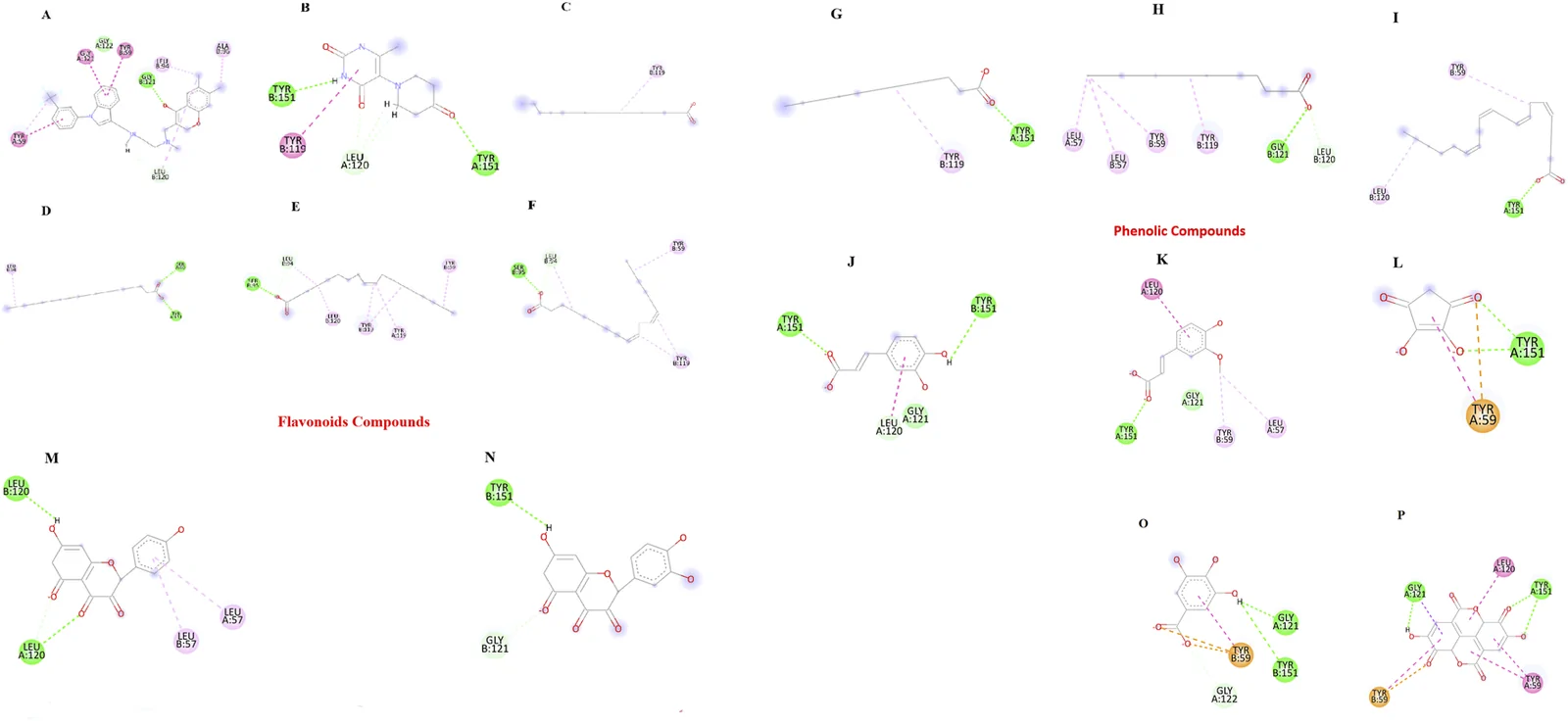
Two-dimensional molecular docking interaction maps illustrating the binding interactions of the co-crystallized ligand and the identified phytochemical constituents with the amino acid residues within the target protein-binding pocket. The colored dashed lines denote the different ligand–protein interactions: bright green, conventional hydrogen bonds; light green, carbon–hydrogen bonds; pink, alkyl and π–alkyl interactions; magenta, π–π interactions; and orange, π-donor hydrogen-bond interactions. The same color-coded interaction key is included within the figure panels for clarity. Panels represent: **(A)** co-crystallized ligand, **(B)** pomalidomide, **(C)** palmitic acid, **(D)** stearic acid, **(E)** oleic acid, **(F)** linoleic acid, **(G)** lauric acid, **(H)** myristic acid, **(I)** arachidonic acid, **(J)** caffeic acid, **(K)** ferulic acid, **(L)** phloroglucinol, **(M)** kaempferol, **(N)** quercetin, **(O)** gallic acid, and **(P)** ellagic acid.

**FIGURE 6:**
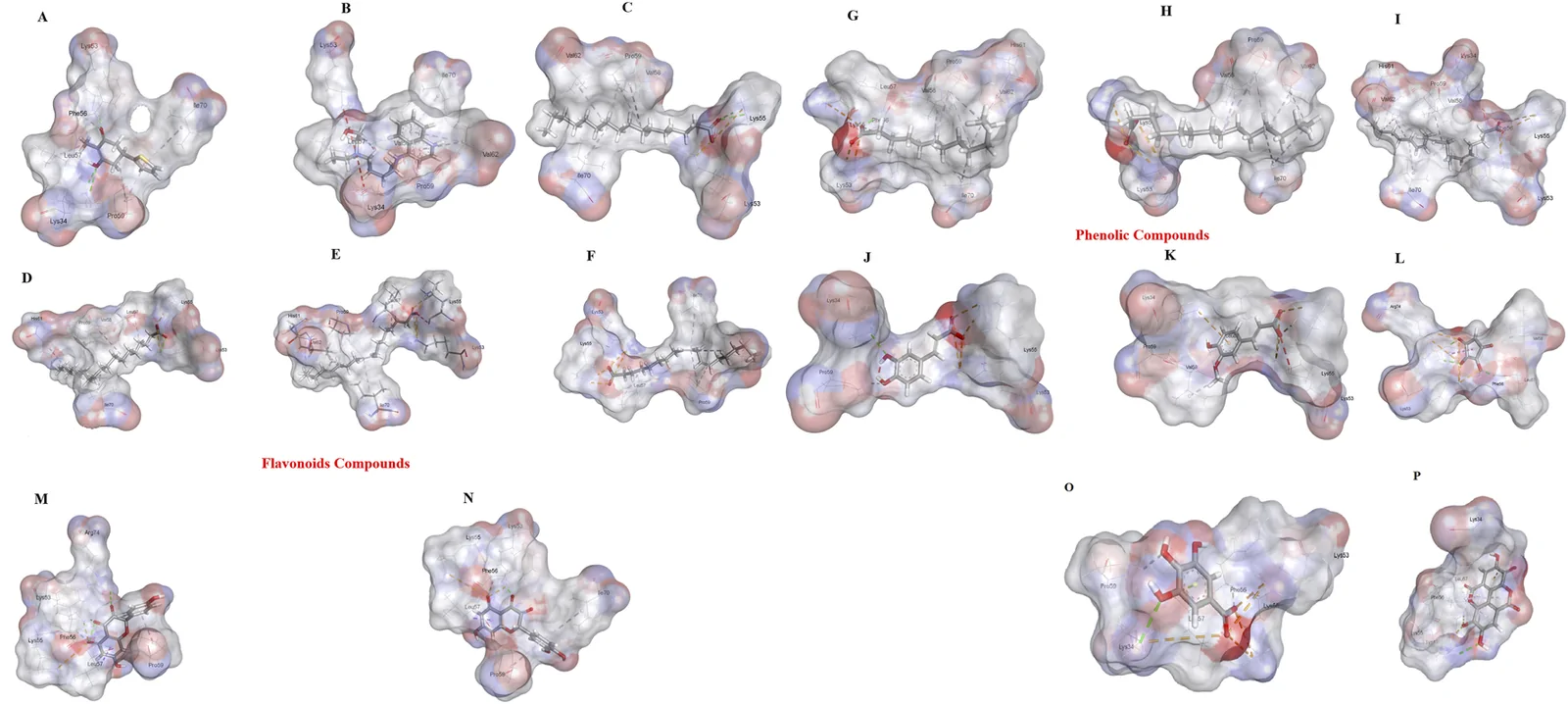
Three-dimensional molecular docking poses showing the binding conformations and molecular-surface interactions of the co-crystallized ligand, reference drug, and identified phytochemical constituents within the active site of the target protein. The protein-binding pocket is displayed as a semi-transparent molecular surface colored according to electrostatic potential, with red, blue, and white regions representing negatively charged, positively charged, and neutral areas, respectively. The docked ligands are shown in stick representation. The colored dashed lines denote the different ligand–protein interactions: green, conventional hydrogen bonds; cyan, carbon–hydrogen bonds; pink, alkyl and π–alkyl interactions; purple, π–π and π–σ interactions; and orange, electrostatic or π-donor hydrogen-bond interactions. Panels correspond to: **(A)** co-crystallized ligand, **(B)** hydroxychloroquine, **(C)** palmitic acid, **(D)** stearic acid, **(E)** oleic acid, **(F)** linoleic acid, **(G)** lauric acid, **(H)** myristic acid, **(I)** arachidonic acid, **(J)** caffeic acid, **(K)** ferulic acid, **(L)** phloroglucinol, **(M)** kaempferol, **(N)** quercetin, **(O)** gallic acid, and **(P)** ellagic acid.

**FIGURE 7:**
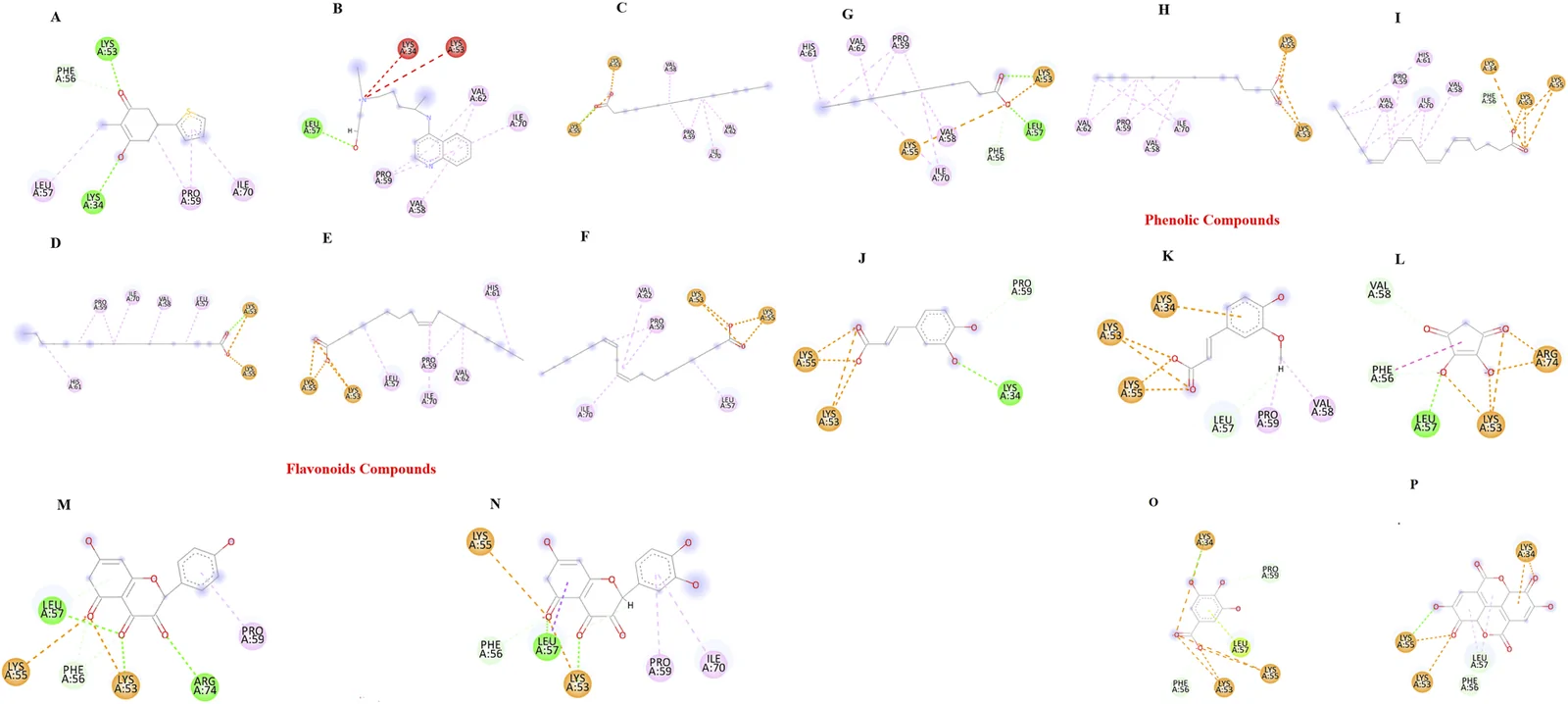
Two-dimensional molecular interaction diagrams illustrating the binding interactions of the co-crystallized ligand, reference drug, and identified phytochemical constituents with amino acid residues within the active site of the target protein. The colored dashed lines denote the different ligand–protein interactions: bright green, conventional hydrogen bonds; light green, carbon–hydrogen bonds; pink, alkyl and π–alkyl interactions; magenta, π–π interactions; purple, π–σ interactions; orange, attractive charge or other electrostatic interactions; and red, unfavorable positive–positive interactions. The corresponding color-coded interaction key is included within the figure panels for clarification. Panels correspond to: **(A)** co-crystallized ligand, **(B)** hydroxychloroquine, **(C)** palmitic acid, **(D)** stearic acid, **(E)** oleic acid, **(F)** linoleic acid, **(G)** lauric acid, **(H)** myristic acid, **(I)** arachidonic acid, **(J)** caffeic acid, **(K)** ferulic acid, **(L)** phloroglucinol, **(M)** kaempferol, **(N)** quercetin, **(O)** gallic acid, and **(P)** ellagic acid.

For LC3B-II, the control hydroxychloroquine showed a docking score of −4.56 kcal mol^−1^. Compared with this docking score, all fatty acids again exhibited stronger predicted binding: palmitic acid scored −5.58 kcal mol^−1^, oleic acid −5.39 kcal mol^−1^, linoleic acid −5.49 kcal mol^−1^, stearic acid −5.42 kcal mol^−1^, lauric acid −5.20 kcal mol^−1^, myristic acid −5.43 kcal mol^−1^, and arachidonic acid −5.86 kcal mol^−1^. These compounds exhibited more favorable predicted docking scores than the reference compound. Among the phenolics, caffeic acid (−4.24 kcal mol^−1^), ferulic acid (−4.40 kcal mol^−1^), and gallic acid (−4.17 kcal mol^−1^) showed weaker binding than hydroxychloroquine, whereas ellagic acid (−5.16 kcal mol^−1^) surpassed the control. The flavonoids kaempferol (−5.05 kcal mol^−1^) and quercetin (−4.97 kcal mol^−1^) also displayed stronger binding than hydroxychloroquine. In conclusion, compared with the references, fatty acids produced more favorable predicted docking scores for both TNF-α and LC3B-II, while only selected phenolics and flavonoids, particularly quercetin, ellagic acid, and kaempferol, achieved better docking scores than the control compounds.

### 3.3 Cytotoxicity against HepG-2 cells and WI-38 normal cells

The algal extract exhibited a clear dose-dependent cytotoxic effect against HepG-2 hepatocellular carcinoma cells after 48 h of treatment (Figure 8). The inhibitory percentage increased progressively with concentration, reaching a maximum inhibition of 96.73% at 1000 µg mL^−1^, while lower concentrations showed a gradual dose-dependent decline in cytotoxic efficiency. IC_50_ and CC_50_ values were determined by nonlinear regression analysis of the dose–response curves using GraphPad Prism (version 9). The calculated IC_50_ for HepG-2 cells was 77.14 ± 1.63 µg mL^−1^, whereas the CC_50_ for WI-38 normal fibroblasts was 141.99 ± 3.54 µg mL^−1^, yielding a selectivity index (SI = CC_50_/IC_50_) of approximately 1.84.

**FIGURE 8:**
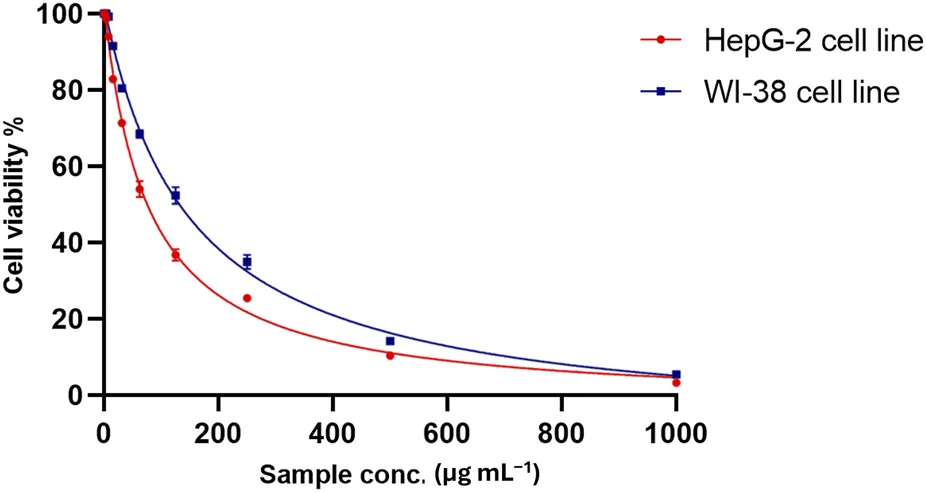
Dose-response curves showing the effect of *H. cuneiformis* extract on HepG-2 and WI-38 cell viability. Data are presented as mean ± SD from three independent biological experiments (n = 3). Curves were fitted using nonlinear regression in GraphPad Prism (version 9), and the fitted curves were used to determine the IC_50_ and CC_50_ values.

### 3.4 Effect of METH and *H. cuneiformis* methanolic extract on hepatic enzyme activities

Figure 9 shows the effects of METH and the algal extract on hepatic enzyme activities. The control group showed normal levels of ALT (19.67 ± 5.85 U L^−1^), and AST (18.33 ± 5.49 U L^−1^) in serum. Rats treated with METH showed marked hepatocellular injury, reflected by significant elevations in serum ALT and AST (89.33 ± 5.59 U L^−1^ and 294.33 ± 5.83 U L^−1^, respectively) compared to control groups. Furthermore, it was observed that ALP activity was also elevated in the METH group (264.67 ± 5.18 U L^−1^) compared to the control group (130.67 ± 5.05 U L^−1^). *H. cuneiformis* administration alone maintained enzyme levels near normal physiological values. Co-treatment with *H. cuneiformis* extract significantly decreased METH-induced liver injury, lowering ALT to 54.33 ± 5.13 U L^−1^, AST to 57.33 ± 4.17 U L^−1^, and ALP to 189.00 ± 5.01 U L^−1^. These results show the hepatoprotective potential of *H. cuneiformis* extract and its capacity to mitigate oxidative and inflammatory liver damage triggered by METH.

**FIGURE 9:**
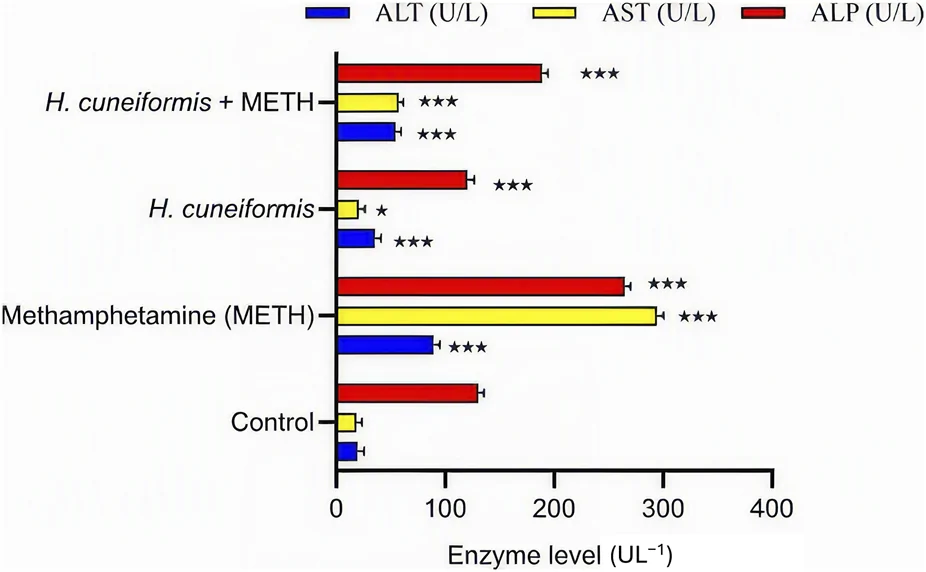
Comparison of serum ALT, AST, and alkaline phosphatase activities among control, METH-treated, algal extract–treated, and combined treatment groups Mean ± SD (n = 8 mice per group).

### 3.5 Western blot analysis

#### 3.5.1 LC3B-II expression

Quantitative densitometric analysis revealed significant inter-group differences in LC3B protein expression (Figure 10; Supplementary Figure S1). METH treatment markedly elevated LC3B levels (0.46 ± 0.04) compared with normal control (0.14 ± 0.03). The algal extract alone did not significantly alter LC3B (0.19 ± 0.03). Co-treatment (METH/ALG) reduced LC3B to 0.22 ± 0.04 vs. METH group (p < 0.05), indicating that *H. cuneiformis* attenuates METH-induced autophagic upregulation.

**FIGURE 10:**
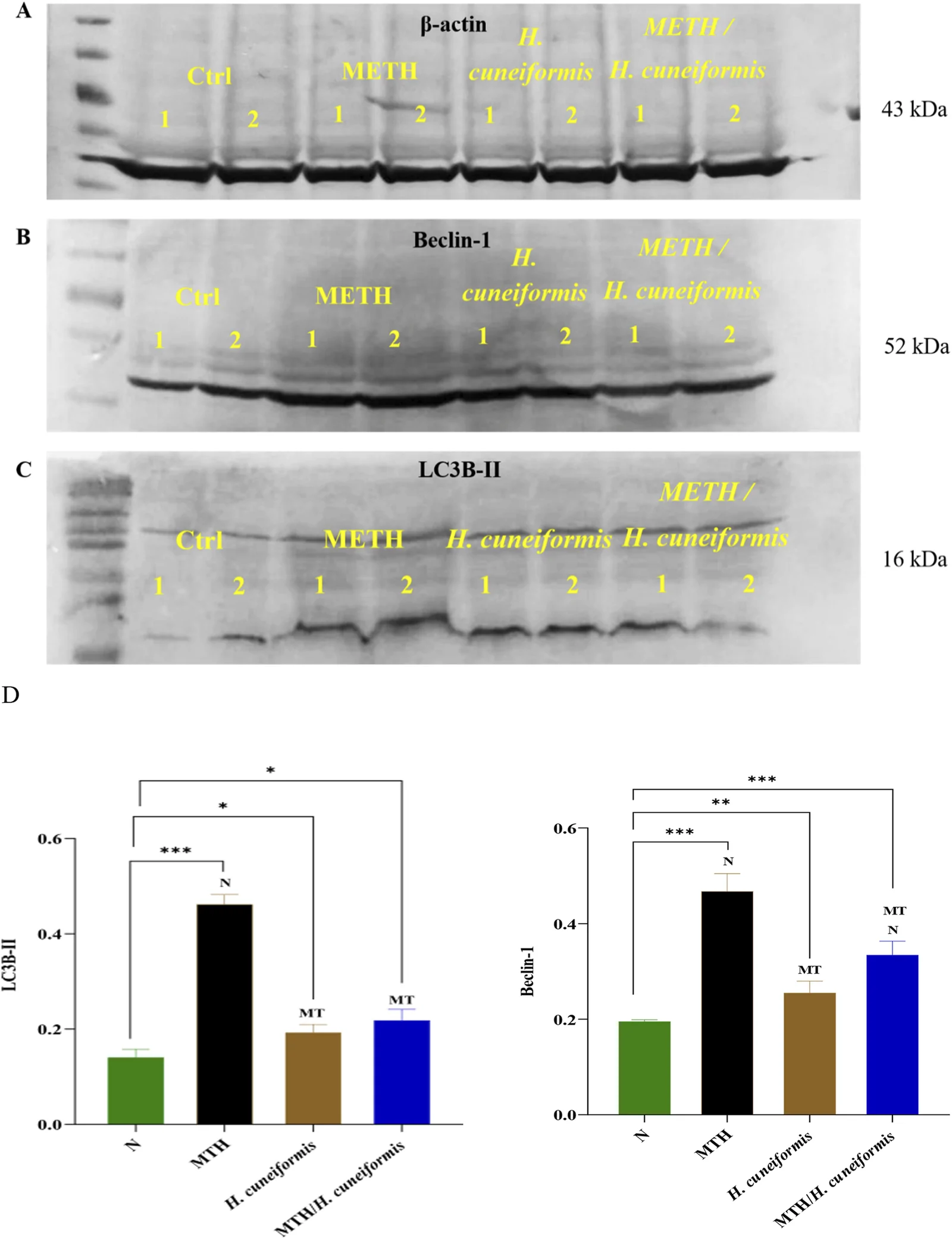
Effects of Hormophysa cuneiformis, methamphetamine (METH), and their combined administration on Beclin-1 and LC3B-II protein expression in liver tissues. **(A)** Representative Western blot bands of β-actin (43 kDa), used as the loading control. **(B)** Representative Western blot bands of Beclin-1 (52 kDa). **(C)** Representative Western blot bands of LC3B‐II (16 kDa). In panels **A–C**, two representative lanes are shown for each experimental group: control, METH, *H. cuneiformis*, and METH/*H. cuneiformis*. **(D)** Densitometric quantification of LC3B-II (left) and Beclin‐1 (right) protein expression normalized to β-actin. Data are presented as mean ± SD (n = 8 mice per group). Statistical significance was determined using one-way ANOVA followed by Tukey‘s post hoc test; *p < 0.05, **p < 0.01, and ***p < 0.001.

#### 3.5.2 Beclin-1 expression

Likewise, Beclin-1 expression was significantly altered among the groups. The control group showed baseline Beclin-1 levels (0.20 ± 0.01). In contrast, METH treatment markedly elevated Beclin-1 expression to 0.47 ± 0.07, indicating strong activation of the autophagic pathway. ALG treatment alone led to a low rise in Beclin-1 levels (0.26 ± 0.05). Particularly, combined METH/*H. cuneiformis* treatment significantly lowered Beclin-1 expression to 0.33 ± 0.06 compared with METH alone, while values remained higher than those of the normal group. This decrease suggests the effect of *H. cuneiformis* on METH-induced autophagy (Figure 10; Supplementary Figure S1).

### 3.6 Histopathological examination

No mortality was recorded in any of the rats during the experimental period. The following results were noticed in almost all sections of all groups.

#### 3.6.1 Hematoxylin and eosin (H&E) staining

The examined liver sections of the control group (Group I) revealed a normal histological structure, consisting of a hexagonal classic hepatic lobule with a central vein and portal tracts at its angles. Hepatocytes were arranged in anastomosing cords one or 2 cells thick, radiating from the center to the periphery, polygonal in shape with acidophilic cytoplasm and central spherical vesicular nuclei with prominent nucleoli; some hepatocytes were binucleated. Numerous mitotic figures were seen, and blood sinusoids lined by flat endothelial cells were present between the hepatocyte cords (Figure 11A).

**FIGURE 11:**
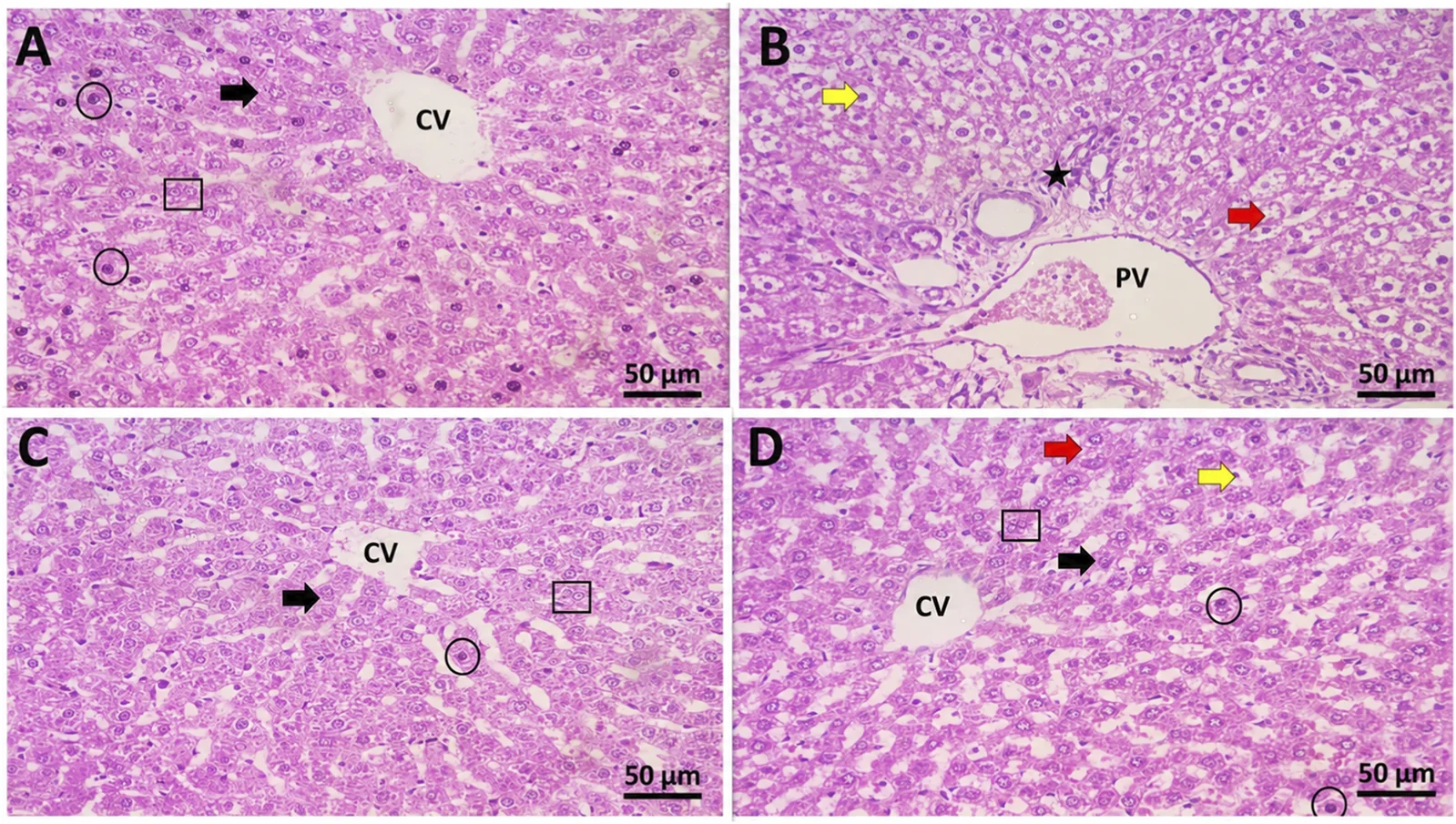
A photomicrograph of sections of the liver of different studied groups; **(A)** control group I showed normal histological architecture consisting of a central vein with radiating cords of normal hepatocytes with moderate acidophilic cytoplasm and central rounded pale-stained nuclei, binucleated hepatocytes, and mitotic figures. **(B)** METH group II showed disrupted histological architecture; dilated portal vein, periportal inflammatory infiltration, marked cytoplasmic vacuolations of hepatocytes and deeply stained shrunken nuclei of numerous hepatocytes. **(C)** *H. cuneiformis* group III revealed normal histological architecture similar to control group. **(D)** METH + *H. cuneiformis* group IV showed marked improvement of histological architecture consisting of central vein with radiating cords of hepatocytes. Most hepatocytes are normal with moderate acidophilic cytoplasm and central rounded pale-stained nuclei (black arrow), while others show cytoplasmic vacuolations and shrunken deeply stained nuclei. Bi nucleated hepatocytes and mitotic figures were seen. (H&E Mic. Mag. x 400) N.B: Central vein (CV), normal hepatocytes (black arrow), binucleated hepatocytes (square), mitotic figure (circle), portal vein (PV), periportal inflammatory infiltration (*), cytoplasmic vacuolations (red arrow) and deeply stained shrunken nuclei (yellow arrow).

Liver sections of Group II (METH) showed marked disruption of this architecture, with dilated, congested portal venules and periportal inflammatory infiltration. Most hepatocytes showed cytoplasmic vacuolation, and others had shrunken, deeply stained nuclei (Figure 11B).

Group III (*H. cuneiformis*) showed histological architecture essentially similar to control, with well-preserved hepatic lobules, normal hepatocyte morphology, and abundant mitotic figures (Figure 11C).

Group IV (METH + *H. cuneiformis*) showed marked improvement relative to Group II, closely resembling control architecture; most hepatocytes appeared normal, though a few still showed shrunken, deeply stained nuclei (Figure 11D).

#### 3.6.2 Caspase-3 and NF-κB immunostaining

Liver sections from the control group showed a negative immune reaction in hepatocytes for both caspase-3 and NF-κB (Figures 12A, 13A). In contrast, the METH group (Group II) exhibited a strong positive immune reaction for both markers (Figures 12B, 13B). The H. cuneiformis-only group (Group III) showed a pattern similar to control, with negative staining in most hepatocytes and only weak positive reactivity in a few cells, for both caspase-3 and NF-κB (Figures 12C, 13C). The combination group (Group IV, METH + *H. cuneiformis*) likewise showed negative or weak positive immunoreactivity for both markers, comparable to control (Figures 12D, 13D). Quantitatively, the mean number of caspase-3- and NF-κB-positive cells was significantly higher in the METH group than in the control and *H. cuneiformis* groups, whereas no significant difference was observed among the control, *H. cuneiformis*, and combination groups (Figure 14).

**FIGURE 12:**
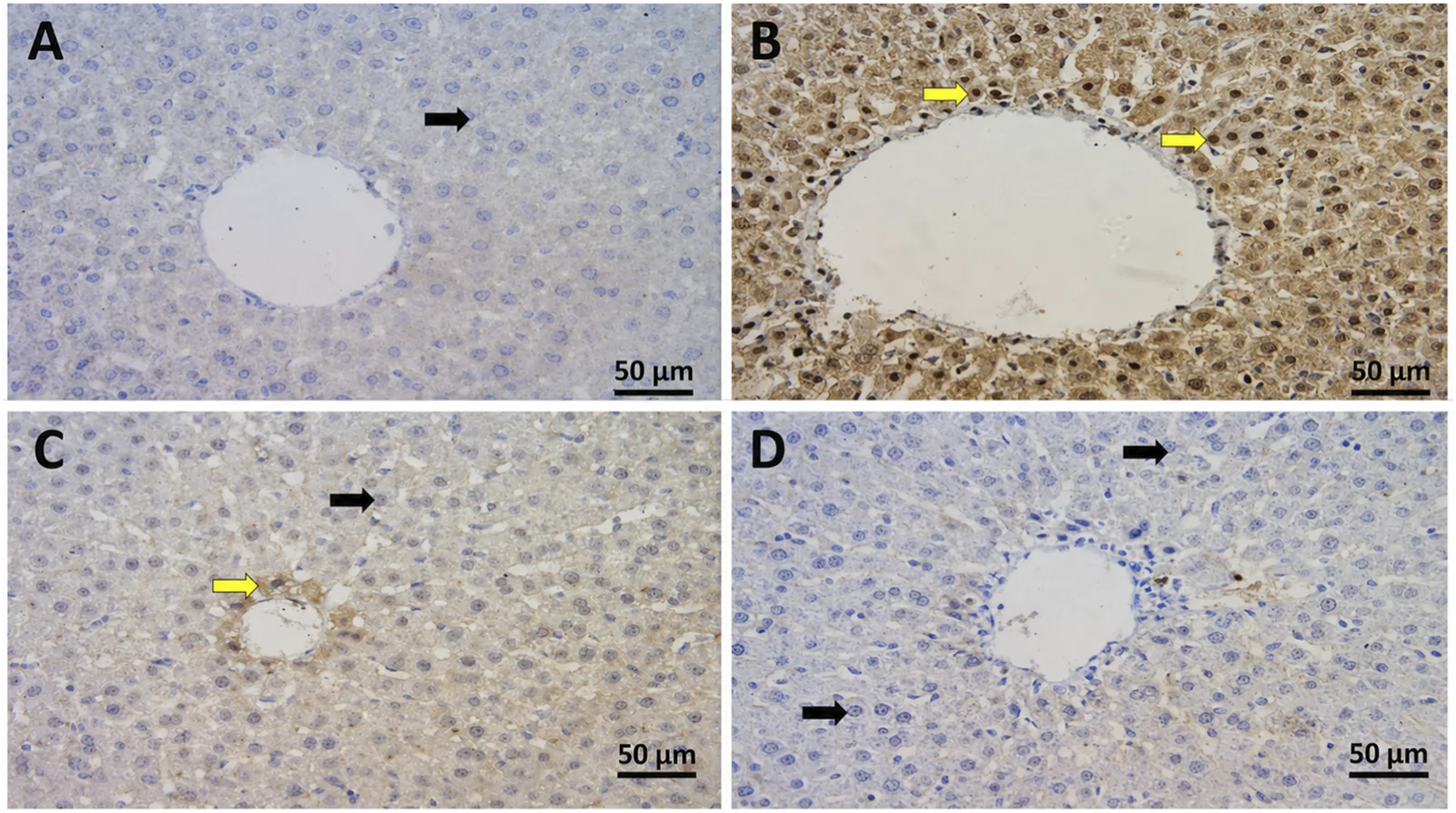
A photomicrograph of a section of liver of different studied groups **(A)** control group showing negative immune reaction in hepatocytes (black arrow). **(B)** group II (Methamphetamine) showing strong positive immune reaction in hepatocytes (yellow arrows) compared to control group. **(C)** group III (*H. cuneiformis)* showing negative reaction in most hepatocytes (black arrow) except for weak positive immune reaction in few hepatocytes (yellow arrow) more or less similar to control group. **(D)** group IV (*H. cuneiformis* + Methamphetamine) showing negative immune reaction in hepatocytes (black arrows) similar to control group. (caspase immunostaining with counterstain with hematoxylin Mic. Mag. x 400).

**FIGURE 13:**
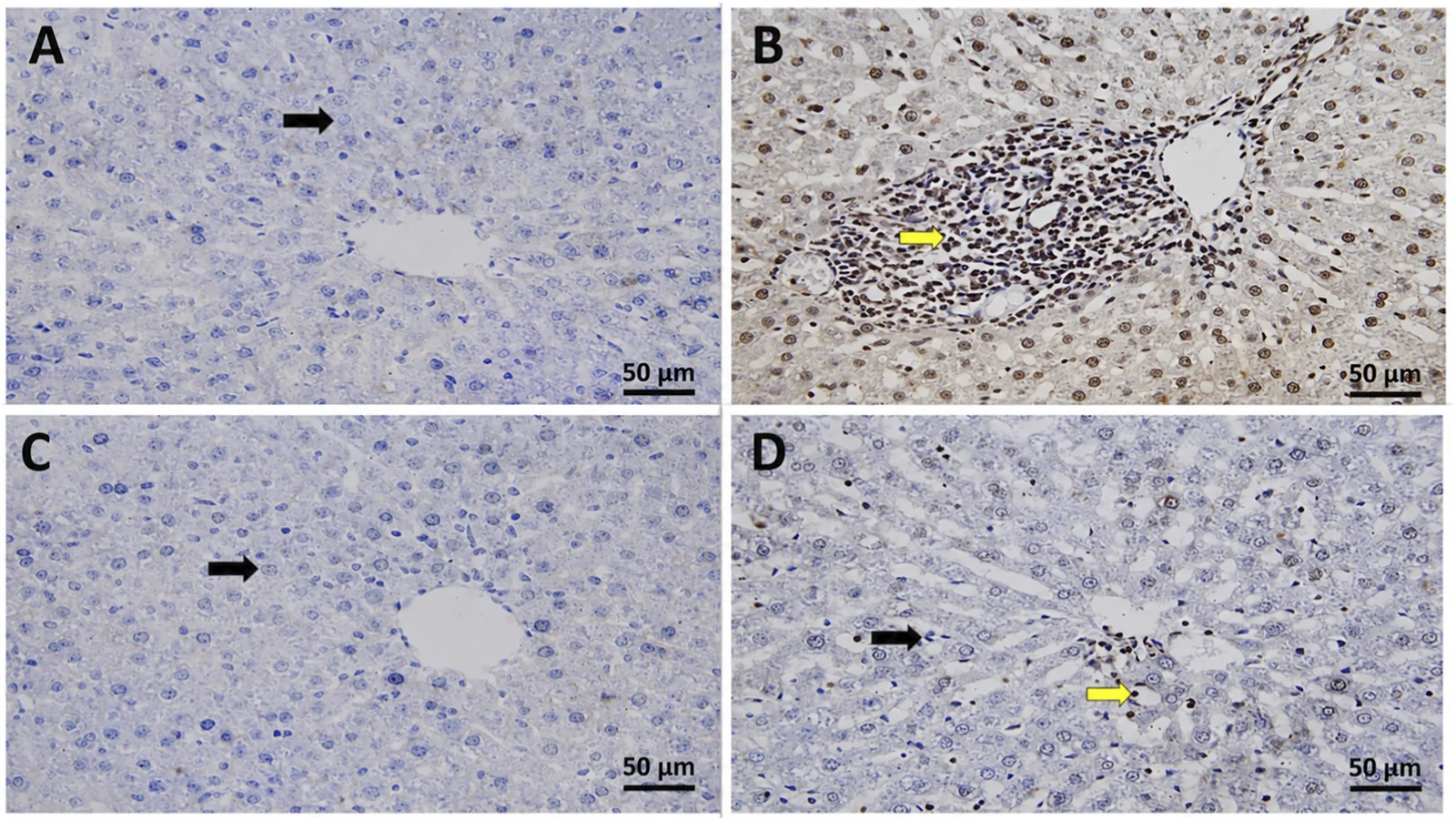
A photomicrograph of a section of liver of different studied groups **(A)** control group showing negative immune reaction (black arrow). **(B)** group II (Methamphetamine) showing strong positive immune reaction (yellow arrows) compared to control group. **(C)** group III (*H. cuneiformis*) showing negative reaction (black arrow) similar to control group. **(D)** group IV (*H*. *cuneiformis* + Methamphetamine) showing weak positive immune reaction in certain area (yellow arrow) and negative reaction in other areas (black arrow) similar to control group. (NF-κB immunostaining with counterstain with hematoxylin Mic. Mag. x 400).

**FIGURE 14:**
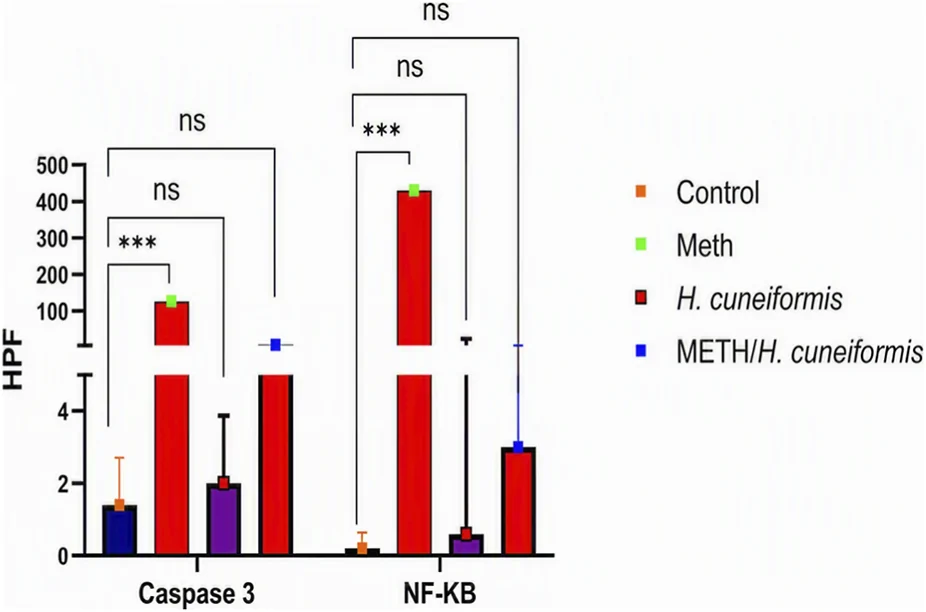
Histogram of the mean number of caspase 3 and NF-κB positive cells of different studied groups.

## 4 Discussion

METH abuse is a growing global health crisis, with the liver representing one of the primary target organs of systemic METH toxicity (Edinoff et al., 2022; Azizi et al., 2023). The present study provides experimental evidence that *H. cuneiformis* confers substantial hepatoprotection against METH-induced liver damage, potentially involving multiple biological processes, including modulation of oxidative stress-related pathways, reduced NF-κB expression, suggesting attenuation of inflammatory signaling, reduced caspase-3 expression, consistent with decreased apoptosis, and modulation of autophagy-related markers. METH administration resulted in marked hepatocellular injury, accompanied by significantly elevated serum ALT, AST, and ALP levels. These results are consistent with previous studies and indicate compromised cell membrane integrity and mitochondrial dysfunction (Ramli et al., 2025). Previous studies have also reported that METH exposure promotes the generation of reactive oxygen species (ROS), induces lipid peroxidation, and disrupts hepatocellular integrity (Omidvari et al., 2023; Adhikary et al., 2026). On the other hand, co-treatment with the algal extract significantly recovered these enzyme levels, indicating preservation of hepatic membrane integrity and restoration of liver function. This biochemical recovery provides strong support for the hepatoprotective potential of the algal extract, which agrees with previous reports emphasizing the protective effects of brown seaweeds-derived bioactive compounds against drug-induced liver injury (Ahmed and Akl, 2025).

The extraction yield varied according to solvent polarity, following the order H_2_O (25.5 mg g^−1^ DW) > methanol (138 mg g^−1^ DW) > ethanol (5.1 mg g^−1^ DW) > hexane (1.5 mg g^−1^ DW). The higher yield obtained with water likely reflects its greater ability to extract hydrophilic constituents, whereas the low yield of hexane is consistent with its selectivity for non-polar compounds. Notably, despite its lower extraction yield, the methanolic extract exhibited the highest antioxidant activity, indicating that extraction yield alone does not necessarily correlate with biological activity and that solvent selection strongly influences the recovery of bioactive metabolites (Holdt and Kraan, 2011; Stengel et al., 2011). Although the aqueous extract contained the highest phenolic content, it displayed lower antioxidant activity than the methanolic extract (Horta et al., 2024), likely because aqueous extraction favors more polar, simpler phenolic compounds with comparatively weaker radical-scavenging capacity (Tofoli et al., 2023), whereas the methanolic extract contains a broader spectrum of bioactive classes alkaloids, saponins, and lipophilic fatty acids/terpenoids identified by GC-MS that may collectively contribute to its antioxidant potential. This suggests that antioxidant activity may depend on the composition and combined effects of bioactive metabolites rather than on total phenolic content alone. The methanolic extract of *H. cuneiformis* demonstrated the strongest antioxidant capacity among the tested solvents, consistent with its high alkaloid, saponin, and fatty acid content, which is consistent with their established antioxidant properties (Abubakar et al., 2024; El-Saadony et al., 2025). The GC–MS analysis identified that *H. cuneiformis* extract is rich in bioactive fatty acids, including oleic, palmitic, linoleic, and arachidonic acids, as well as terpenoids and sterol derivatives. These compound classes have been associated with antioxidant, anti-inflammatory, and membrane-stabilizing properties. However, because the crude methanolic extract was not fractionated and its individual constituents were not evaluated separately, the relative contribution of each compound class to the observed antioxidant activity cannot be conclusively established. Future studies employing bioactivity-guided fractionation are therefore required to identify the principal antioxidant constituents.

Phenolic compounds were identified as a major class of secondary metabolites in *H. cuneiformis*, particularly in the aqueous extract. These phenolics were further evaluated through molecular docking analysis. Among the tested compounds, caffeic acid, ferulic acid, phloroglucinol, gallic acid, and ellagic acid showed weaker binding affinities compared with the reference control. In contrast, the flavonoids kaempferol and quercetin also displayed stronger binding than hydroxychloroquine. Collectively, quercetin and phlorotannin demonstrated superior binding compared to the control, while all tested fatty acids demonstrated potential interaction affinities. These findings are consistent with the previous studies which demonstrated that phenolic compounds inside *H. cuneiformis* exhibit strong antioxidant and anti-inflammatory activities, including inhibition of lipid peroxidation and NF-KB mediated inflammation (Ahmed and Akl, 2025). Dysregulated autophagy is observed by following the expression levels of LC3B-II and Beclin-1 after treatment with METH. The marked upregulation of LC3B-II and Beclin-1 observed in METH-treated rats suggests excessive autophagic activation, a process closely associated with hepatocyte death and inflammation. LC3B-II accumulation may reflect enhanced autophagosome formation; however, further flux analysis would clarify autophagic turnover (du Toit et al., 2018; Liebl et al., 2022). Particularly, co-administration of *H. cuneiformis* extract significantly reduced the expression of both markers, suggesting modulation of autophagy-related responses. However, because autophagic flux and additional markers such as p62/SQSTM1 (Larsen et al., 2010) were not evaluated, these findings should be interpreted as supportive rather than definitive evidence of autophagy regulation. These findings are in line with previous reports demonstrating that algal antioxidants exhibited protective behavior in modulation of pathological autophagy through the suppression of oxidative stress and pro-inflammatory signaling pathways (Vignaud et al., 2023; Matin et al., 2024). Histopathological analysis further supported the biochemical findings. Liver sections from METH-treated rats showed obvious structural disruption, including hepatocellular vacuolation, nuclear pyknosis, vascular congestion, and inflammatory cell infiltration. These pathological characteristics are typical of oxidative stress–driven hepatocellular degeneration and have been consistently documented in experimental models of METH-induced liver toxicity (Eskandari et al., 2014). It should be noted that the present study did not directly assess indicators of hepatic lipid or glucose metabolism, nor did it measure mitochondrial function; the vacuolar degeneration observed histologically may reflect several underlying processes and cannot, on its own, confirm steatosis or a specific metabolic mechanism. Other studies have reported that METH exposure can alter hepatic lipid, protein, and carbohydrate metabolism and impair glucose and lipid homeostasis, potentially contributing to hepatic steatosis and mitochondrial dysfunction (Ramli et al., 2025; Eskandari et al., 2014). Based on these prior reports, and supported by the elevated caspase-3 expression observed in the present study, it is plausible that mitochondrial-associated apoptotic signaling may partly underlie the histopathological changes observed here; however, because mitochondrial function and lipid and glucose metabolic indicators were not directly measured in this work, this remains a hypothesis for future investigation rather than a conclusion of the present study. On the other hand, co-treatment with *H. cuneiformis* largely preserved normal hepatic architecture, with only mild residual changes observed, indicating substantial structural recovery. Moreover, the absence of histopathological abnormalities in the *H. cuneiformis* -only group showed no apparent histopathological toxicity at the administered dose under the present experimental conditions. These results support the protective behavior of *H. cuneiformis*.

Immunohistochemical staining offered additional evidence for the protective action of *H. cuneiformis*. METH treatment was associated with a noticeable increase in caspase-3 positive hepatocytes, indicating enhanced apoptotic cell death in hepatic tissue. This observation is in line with earlier studies reporting that METH-induced hepatotoxicity involves activation of the intrinsic mitochondrial apoptotic pathway (Zeng et al., 2022; Ramli et al., 2025). On the other hand, co-administration of the *H. cuneiformis* extract significantly reduced caspase expression, consistent with an anti-apoptotic effect. This finding is supported by previous studies demonstrating that bioactive compounds from algae can inhibit mitochondrial-mediated apoptosis through their antioxidant and anti-inflammatory actions (Agena et al., 2023; Sharma et al., 2023).

Liver inflammation plays a main role in inducing METH-related hepatotoxicity, along with NF-κB, which has a main role in monitoring of inflammatory gene activity. In the present study, immunohistochemical analysis revealed a significant elevation in NF-κB -positive hepatocytes in the METH-treated group compared with controls (Shi et al., 2025; Weiselberg et al., 2025), confirming that METH exposure drives NF-κB activation, pro-inflammatory cytokine release, and subsequent hepatic inflammation. On the other hand, co-treatment with *H. cuneiformis* extensively decreased NF-κB expression, suggesting attenuation of inflammatory signaling. Polyphenols, saponins, and fatty acids in the algal extract drive the anti-inflammatory effect by inhibiting NF-κB–mediated signaling (Cadar et al., 2025; Mathanmohun et al., 2025). Molecular docking analysis further predicted favorable binding scores of these fatty acids toward TNF-α and LC3B-II, compared to reference inhibitors. These *in silico* findings provide supportive, hypothesis-generating evidence that fatty acids present in *H. cuneiformis* may contribute to the observed modulation of inflammatory and autophagy-related pathways. However, considering the chemical complexity of the extract, the potential contributions of other bioactive constituents cannot be excluded. Collectively, these findings suggest that *H. cuneiformis* exerts hepatoprotective effects against METH-induced liver injury and that these effects may be associated with modulation of oxidative stress-related pathways, attenuation of NF-κB-associated inflammatory responses, reduced apoptosis, and regulation of autophagy-related markers. However, the present study has several limitations. Although our biochemical, histopathological, immunohistochemical, and molecular findings provide supportive evidence for these biological processes, they do not constitute definitive mechanistic proof. Direct assessment of oxidative stress biomarkers (e.g., malondialdehyde (MDA), reduced glutathione (GSH), superoxide dismutase (SOD), and catalase) and additional autophagy markers such as p62/SQSTM1 or autophagic flux was not performed. Future studies incorporating these complementary biomarkers and functional mechanistic analyses are warranted to validate the molecular pathways underlying the hepatoprotective effects of *H. cuneiformis*. An additional limitation relates to the experimental design: the METH group (Group II) was sacrificed after 7 days, whereas the control, extract-only, and combination groups were maintained for 10 days. This shorter METH exposure window was deliberately selected to model an early, minimum hepatotoxic dose rather than a maximal-injury timepoint, following established protocols (Jing et al., 2014; Zhang et al., 2022); however, a contribution of differential housing duration to the observed group differences cannot be fully excluded, and future studies should adopt a fully time-matched design to formally rule out this potential confound. Another limitation of this study is that cytotoxicity was assessed using WI-38 human lung fibroblasts rather than normal hepatocytes. Although WI-38 cells provide a useful initial safety model, future studies employing normal hepatic cell lines (e.g., L02 or THLE-2) are warranted to further confirm the liver-specific safety of the extract.

## 5 Conclusion

Natural biological sources, particularly brown seaweeds, are promising resources for protecting against liver toxicity. In this study, *H. cuneiformis* extract showed significant protection against methamphetamine (METH)-induced liver injury through multiple mechanisms. METH exposure caused severe hepatotoxicity, characterized by elevated liver enzymes, oxidative stress, excessive autophagy, inflammation, apoptosis, and histopathological damage. Co-administration of the extract normalized enzyme levels, maintained hepatic architecture, and alleviated both molecular and cellular alterations. The hepatoprotective effects are largely attributed to the extract’s strong antioxidant capacity and rich phytochemical composition, including phenolics, flavonoids, saponins and bioactive fatty acids. These compounds suppressed NF-κB–mediated inflammatory responses, inhibited caspase-dependent apoptosis, and regulated dysregulated autophagy, as evidenced by the normalization of LC3B-II and Beclin-1 expression. Furthermore, molecular docking analysis revealed strong binding affinities between algal constituents and key molecular targets associated with inflammatory signaling and autophagy regulation. Overall, *H. cuneiformis* represents a scientifically promising, naturally derived hepatoprotective candidate whose multi-target pharmacological profile spanning antioxidant, anti-inflammatory, anti-apoptotic, and autophagy-regulatory activities warrants further preclinical and translational investigation, particularly in the context of substance-abuse-related organ injury.

## Data Availability

The original contributions presented in the study are included in the article/Supplementary Material, further inquiries can be directed to the corresponding author.
