# Supplementary material for: Hormophysa cuneiformis protects against methamphetamine-induced hepatotoxicity via antioxidant, anti-inflammatory, and autophagy-regulatory mechanisms: histological and biochemical studies
Source: Front Pharmacol. 2026 Sep 14;17:1913493. doi: 10.3389/fphar.2026.1913493 (PMC13616779; doi:10.3389/fphar.2026.1913493)
Supplement: Supplementary file 1 [file DataSheet1.pdf]

1- **Beta actin**

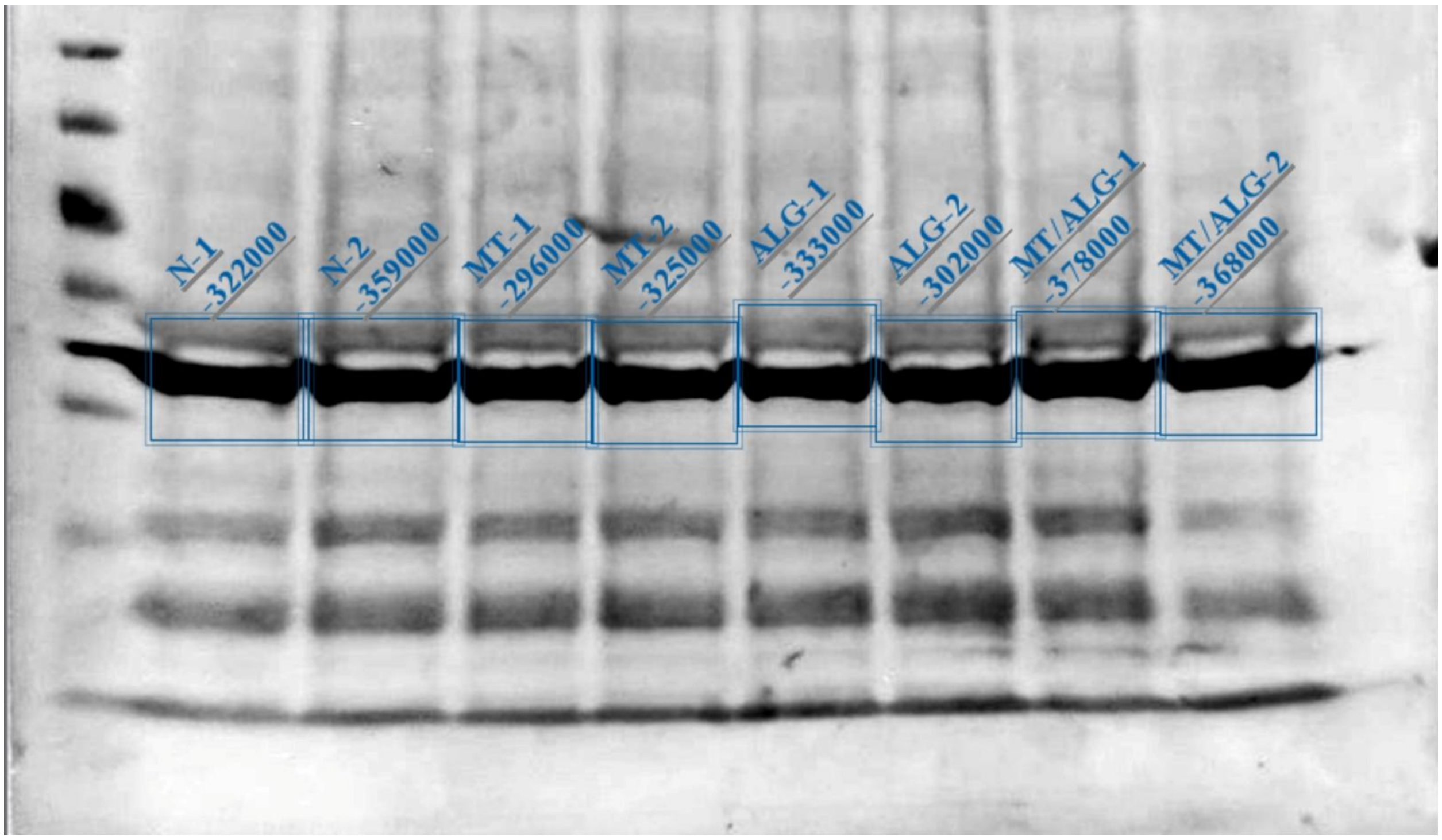
N-1
-322000
N-2
-359000
MT-1
-296000
MT-2
-325000
ALG-1
-333000
ALG-2
-302000
MT/ALG-1
-378000
MT/ALG-2
-368000

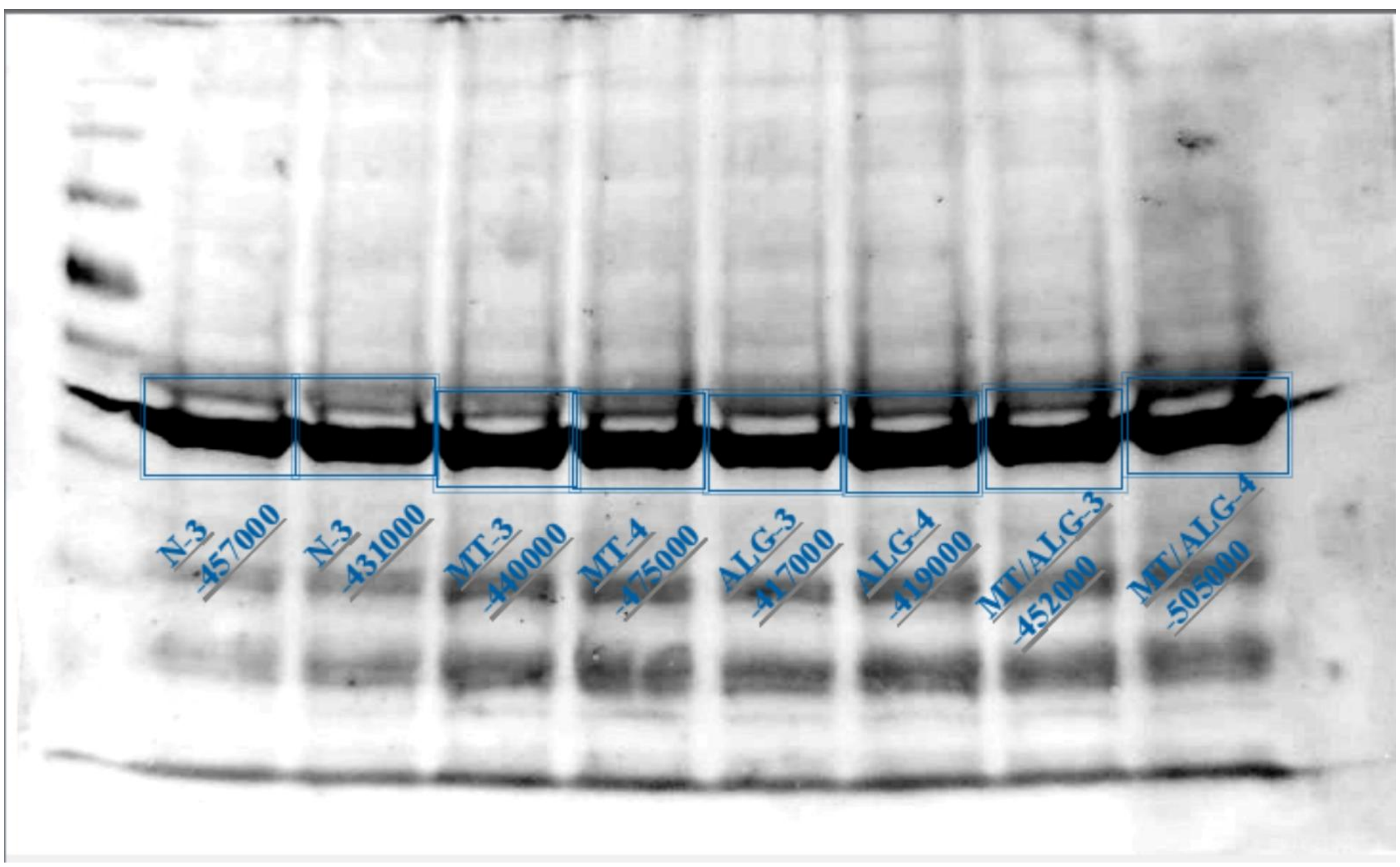
N-3
-457000
N-3
-431000
MT-3
-440000
MT-4
-475000
ALG-3
-417000
ALG-4
-419000
MT/ALG-3
-452000
MT/ALG-4
-505000

2- Beclin-1

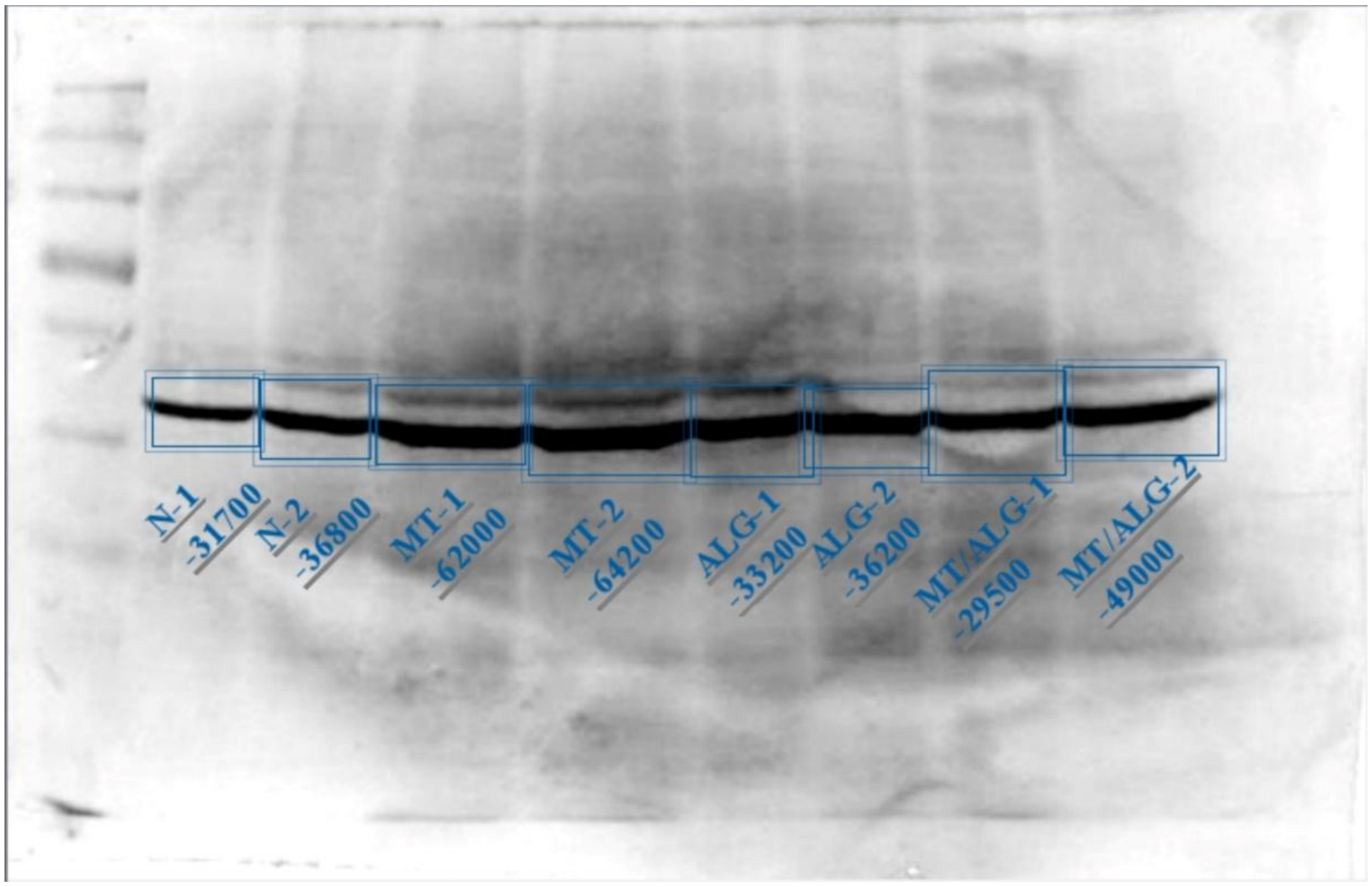

N-1
-31700
N-2
-36800
MT-1
-62000
MT-2
-64200
ALG-1
-33200
ALG-2
-36200
MT/ALG-1
-29500
MT/ALG-2
-49000

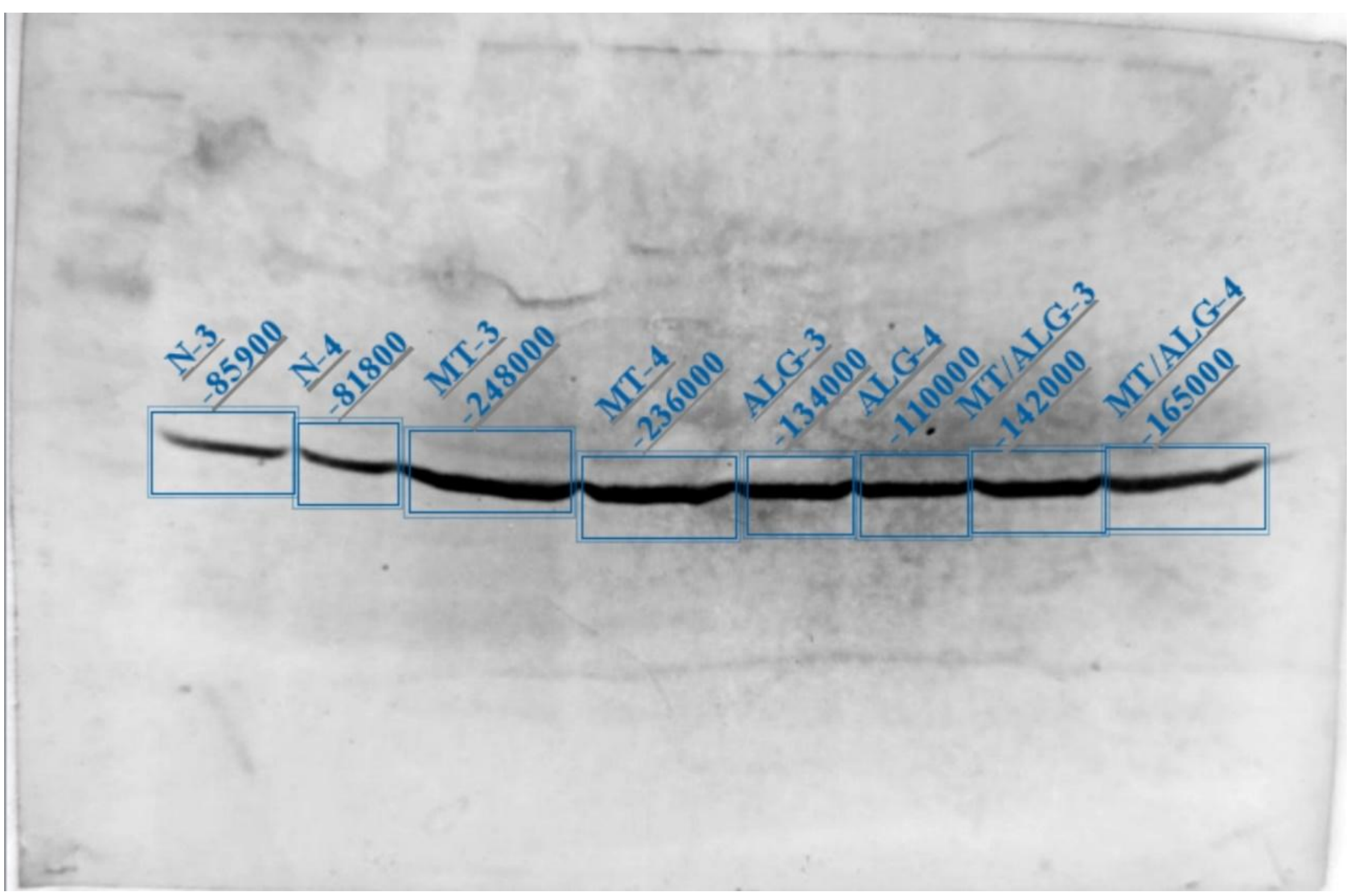

N-3
-85900
N-4
-81800
MT-3
-248000
MT-4
-236000
ALG-3
-134000
ALG-4
-110000
MT/ALG-3
-142000
MT/ALG-4
-165000

3- **LC3B**

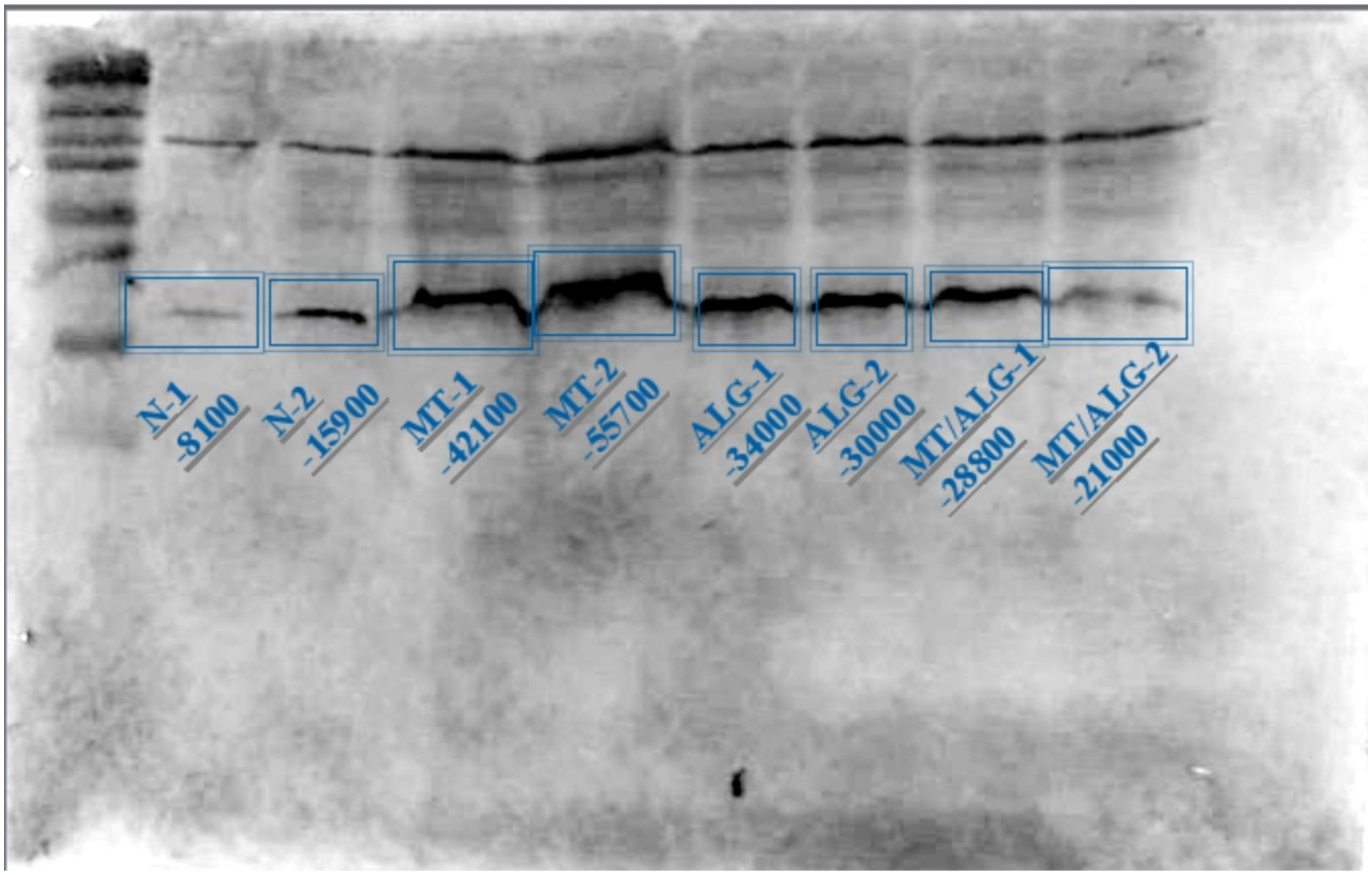
N-1
-8100
N-2
-15900
MT-1
-42100
MT-2
-55700
ALG-1
-34000
ALG-2
-30000
MT/ALG-1
-28800
MT/ALG-2
-21000

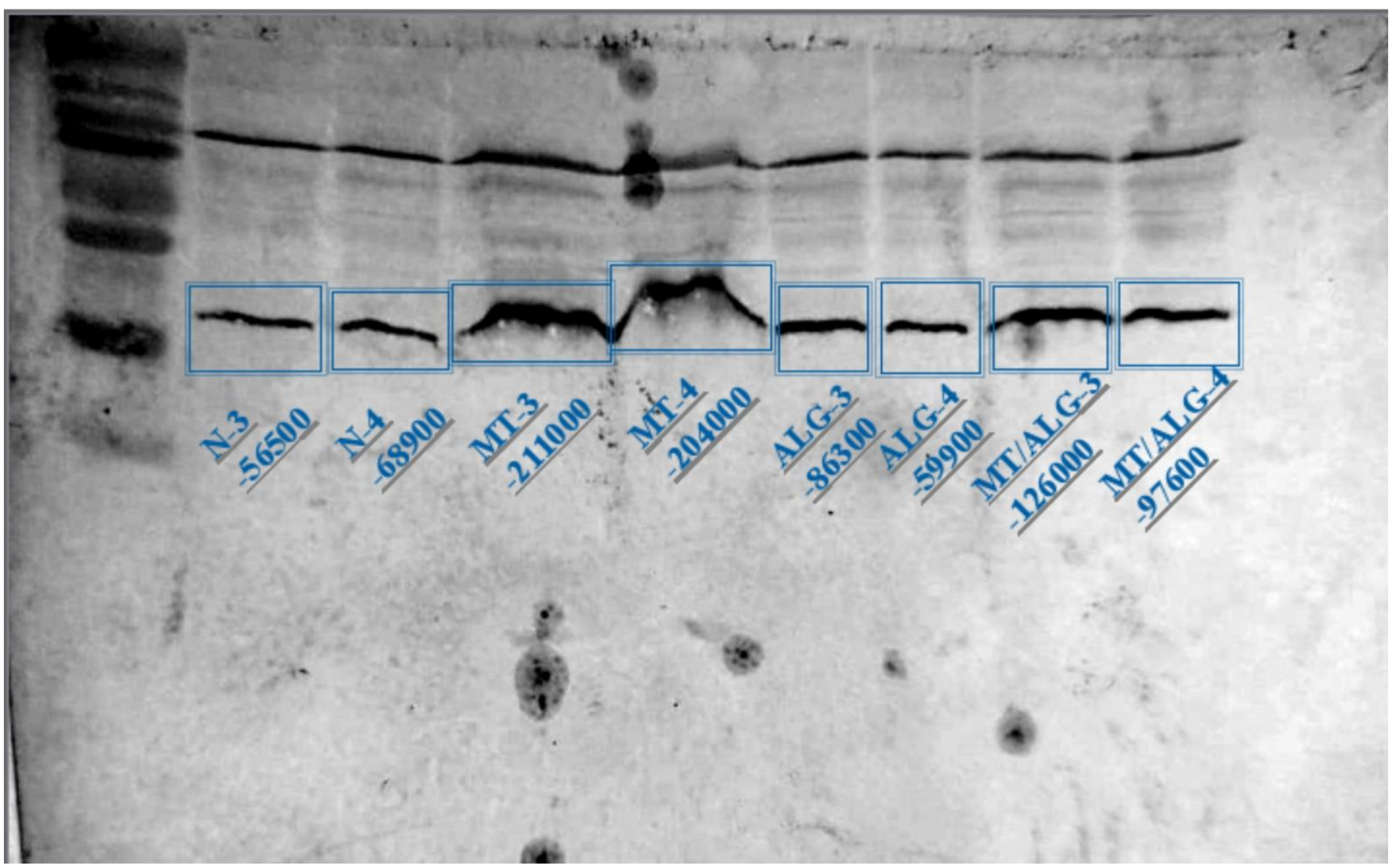
N-3
-56500
N-4
-68900
MT-3
-211000
MT-4
-204000
ALG-3
-86300
ALG-4
-59900
MT/ALG-3
-126000
MT/ALG-4
-97600

Figure S1: Effects of *H. cuneiformis*, methamphetamine (METH), and combined METH/*H. cuneiformis* treatment on Beclin-1 and LC3B-II protein expression in liver tissues, as determined by Western blot analysis, normalized to β-actin.
